# Unveiling the hub genes in the SIGLECs family in colon adenocarcinoma with machine learning

**DOI:** 10.3389/fgene.2024.1375100

**Published:** 2024-04-08

**Authors:** Tiantian Li, Ji Yao

**Affiliations:** ^1^ Key Laboratory of Systems Biomedicine (Ministry of Education), Shanghai Center for Systems Biomedicine, Shanghai Jiao Tong University, Shanghai, China; ^2^ Department of Astronomy, School of Physics and Astronomy, Shanghai Jiao Tong University, Shanghai, China; ^3^ Shanghai Astronomical Observatory, Shanghai, China

**Keywords:** colon adenocarcinoma, bioinformatics, SIGLEC, principal component analysis, self-organizing map

## Abstract

**Background:**

Despite the recognized roles of Sialic acid-binding Ig-like lectins (SIGLECs) in endocytosis and immune regulation across cancers, their molecular intricacies in colon adenocarcinoma (COAD) are underexplored. Meanwhile, the complicated interactions between different SIGLECs are also crucial but open questions.

**Methods:**

We investigate the correlation between SIGLECs and various properties, including cancer status, prognosis, clinical features, functional enrichment, immune cell abundances, immune checkpoints, pathways, etc. To fully understand the behavior of multiple SIGLECs’ co-evolution and subtract its leading effect, we additionally apply three unsupervised machine learning algorithms, namely, Principal Component Analysis (PCA), Self-Organizing Maps (SOM), K-means, and two supervised learning algorithms, Least Absolute Shrinkage and Selection Operator (LASSO) and neural network (NN).

**Results:**

We find significantly lower expression levels in COAD samples, together with a systematic enhancement in the correlations between distinct SIGLECs. We demonstrate SIGLEC14 significantly affects the Overall Survival (OS) according to the Hazzard ratio, while using PCA further enhances the sensitivity to both OS and Disease Free Interval (DFI). We find any single SIGLEC is uncorrelated to the cancer stages, which can be significantly improved by using PCA. We further identify SIGLEC-1,15 and CD22 as hub genes in COAD through Differentially Expressed Genes (DEGs), which is consistent with our PCA-identified key components PC-1,2,5 considering both the correlation with cancer status and immune cell abundance. As an extension, we use SOM for the visualization of the SIGLECs and show the similarities and differences between COAD patients. SOM can also help us define subsamples according to the SIGLECs status, with corresponding changes in both immune cells and cancer T-stage, for instance.

**Conclusion:**

We conclude SIGLEC-1,15 and CD22 as the most promising hub genes in the SIGLECs family in treating COAD. PCA offers significant enhancement in the prognosis and clinical analyses, while using SOM further unveils the transition phases or potential subtypes of COAD.

## 1 Introduction

Colorectal cancer is a major worldwide cancer, making up 10% of all cancer cases each year (Ionescu et al., 2023). In the category of colorectal cancers, COAD is noteworthy, ranking as the second most common in women and the third most common in men (Bajramagic et al., 2019; Sung et al., 2021). The prognosis of COAD depends on its stage. Late-stage and metastatic cases usually have less favorable outcomes (Siegel et al., 2020). Prognostic factors include how well drugs work and the chance of cancer coming back after surgery (Martini et al., 2020). Hence, early and accurate diagnosis is crucial for effective COAD management.

In recent years, the clinical efficacy of immune checkpoint inhibitors, such as PD-1, PD-L1, and CTLA-4, has been demonstrated in COAD treatment (Le et al., 2017; Cantero-Cid et al., 2018; Johdi et al., 2020). Noteworthy drugs in this category, including Opdivo (nivolumab), Keytruda (pembrolizumab), and Yervoy (ipilimumab), have been employed in clinical trials (Boland et al., 2017). Concurrently, targeted therapies such as panitumumab and cetuximab, which focus on EGFR, have become important in treating metastatic colon cancer, opening a new era in COAD treatment (Ketzer et al., 2018). Despite these advancements, the 5-year survival rate for COAD patients is still unsatisfactory (Lichtenstern et al., 2020). Changes in gene expression are crucial in the development of COAD. Therefore, it is essential to conduct additional research on COAD-related genes and their underlying mechanisms to advance future diagnostic and treatment strategies.

SIGLECs, also known as Sialic acid-binding Ig-like lectins, constitute essential components of the immunoglobulin superfamily (IgSF) and are exclusively expressed in immune cells (Macauley et al., 2014). Presently, 14 human subtypes and 9 mouse subtypes have been identified (Duan et al., 2020). These SIGLECs play a crucial role in modulating immune responses and interactions among immune cells by binding primarily to sialic acid residues on cell surfaces. They regulate various physiological processes, including immune cell activation, apoptosis, adhesion, and inflammatory responses. Additionally, SIGLECs are pivotal in immune tolerance, autoimmune diseases, and immune evasion. Targeting SIGLECs holds promising implications for advancing treatment strategies for immune-related diseases. Furthermore, SIGLECs bind to specific ligands in the tumor microenvironment, exerting significant influence on various physiological and pathological processes within tumors (Macauley et al., 2014; van Houtum et al., 2021). Notably, inhibitory SIGLECs, such as CD33, SIGLEC-5, 7, 9, and 10, are upregulated in tumor-infiltrating CD4 and CD8 T cells across various cancer types, contributing to the failure of T cell responses (Jiang et al., 2022). While tumor-associated macrophages (TAM) traditionally activate immune responses through the phagocytosis of damaged cancer cells (Murray et al., 2014; Noy et al., 2014; Nahrendorf et al., 2016), in specific cases, SIGLEC10 on macrophages can engage with CD24 expressed on breast cancer cells, initiating a protective immune response and hindering cancer cell phagocytosis (Barkal et al., 2019; DeNardo et al., 2019).

A large amount of evidence suggests that SIGLECs receptors hold promise as novel immune checkpoints and attractive targets for cancer immunotherapy (Lim et al., 2021; Stanczak et al., 2023). Research suggests that combining SIGLEC15 with PD-L1 antagonists holds promise for treating colorectal cancer (Ahmad et al., 2023). SIGLEC7 is implicated in modulating the immune response to *Fusobacterium nucleatum* in colorectal cancer (Lamprinaki et al., 2021). Furthermore, SIGLEC6 may be associated with mast cell activity in the tumor microenvironment of colorectal cancer (Yu et al., 2018). Additionally, SIGLEC15 demonstrates immunosuppressive effects in pre-metastatic lymph nodes of colorectal cancer, presenting a potential target for immunotherapy (Du et al., 2021). However, our understanding of the specific mechanisms involving SIGLECs in the initiation and progression of COAD is currently limited. Furthermore, comprehensive reports addressing the expression of SIGLECs in COAD and their correlation with immune molecules are conspicuously absent in the existing literature. This underscores the need for further research to unveil the intricate role of SIGLECs in COAD, offering potential avenues for therapeutic intervention in cancer immunotherapy.

In this study, our approach involves using a public database alongside various statistical tools to meticulously examine the variations in SIGLEC expressions among patients with COAD. Specifically, we explore the statistical correlation between each SIGLEC’s expression and the cancer status through 1-dimensional correlation analysis (1-D correlation). Additionally, we delve into the dynamic changes in correlation between different SIGLECs (2-D correlation) and the simultaneous alterations in all SIGLECs (n-D correlation) using five machine-learning algorithms, including the Principal Component Analysis (PCA), the neural network (NN), the Self-Organizing Maps (SOM), Least Absolute Shrinkage and Selection Operator (LASSO) and K-means.

Expanding our analysis, we delve into clinical data to unravel the roles of SIGLECs in relation to general clinical features, including survival curves, Hazzard ratio, various cancer stages, gender, age, etc. To gain a deeper understanding of the mechanisms underlying the interactions that SIGLECs contribute to, our investigation encompasses difference analysis, Gene Ontology (GO) and Kyoto Encyclopedia of Genes and Genomes (KEGG) Analyses, Protein-Protein Interaction-Network (PPI-Network) exploration, and scrutiny of the immune-cell landscape. The PCA algorithm is applied in addition to general SIGLEC analysis to disentangle their complicated interactions and identify the major contributions. The SOM algorithm is used for visualizations and feature subtractions to demonstrate the co-evolution with other properties, i.e., immune cells and cancer stages. Other machine learning algorithms such as NN, LASSO and K-means work as supporting tools for corresponding sub-topics. This multi-faceted approach aims to provide a comprehensive and nuanced insight into the intricate landscape of SIGLEC expressions in COAD patients, shedding light on their potential roles in clinical outcomes, and underlying molecular and immune processes.

## 2 Materials and methods

### 2.1 Data collection

The datasets utilized in this study were sourced from two reputable repositories. The TCGA datasets, spanning 20 types of cancer, including the TCGA-COAD dataset, were acquired from the TCGA database (https://portal.gdc.cancer.gov/). Additionally, the COAD-related RNA sequencing dataset GSE110224 was retrieved from the GEO database (https://www.ncbi.nlm.nih.gov/geo/). The TCGA-COAD include 453 COAD and 41 normal samples. This dataset not only included RNA sequencing data but also incorporated valuable survival and clinical information for a thorough analysis. Concurrently, the GSE110224 dataset was employed as the validation dataset, featuring 17 samples each, for COAD and normal. The GSE39582 dataset was also collected, with 19 normal samples and 566 cancer samples.

### 2.2 PCA of SIGLECs in COAD

PCA is a very powerful tool for subtracting key features, de-noising, and dimensionality-reduction in a wide field of data sciences. It performs a linear transformation of high-dimensional data and results in a set of orthogonal PCs. For the detailed methodology, we refer to some literatures for your interest (Ma et al., 2011; Lever et al., 2017).

We first use the “sklearn” python package (https://scikit-learn.org/stable/index.html) to rescale the 14 SIGLECs expression data into standard Gaussian distributions and build a resulting covariance matrix. Then “numpy” package (https://numpy.org/) is used to calculate the eigen values and eigen vectors, which directly give the projection matrix that linearly transforms the 14 SIGLECs into 14 PCs.

### 2.3 Prognostic analysis

The prognostic significance of SIGLECs in COAD was evaluated through univariate Cox regression (uniCox) and Kaplan-Meier (K-M) survival analyses (Liu et al., 2018). The Overall survival (OS) and disease-free interval (DFI) of COAD patients with differential expression of SIGLECs were evaluated using the “survminer” and “survival” R package. COAD patients were divided into low and high expression groups based on the median expression level of SIGLECs, while validations with different binning methods are also provided in the SI.

In addition to the univariate Cox regression, to account for the complicated biological interactions and co-evolutions, we perform a multivariate Cox regression, on top of the previous PCA analysis as well as a Least Absolute Shrinkage and Selection Operator (LASSO) regression. The LASSO regression is calculated with the “glmnet” R package.

### 2.4 Neural network (NN)

We use the “neuralnet” R package to build a neural network as a supporting tool, to show the sensitivity and promising capability of machine learning in the future with large dataset. We use two hidden layers of (8,2) neurons, with the small number of neurons aiming to prevent overfitting, and enough depth of the network to account for potential non-linearities in the data. We randomly select half of the patients as training sample and use the other half as testing sample, to prevent bias due to train/test imbalance as the number of patients at risk is low. We train the network for 3,000 epochs with learning rate 0.01 to prevent overfitting. Visualizations of the NN is done with the “NeuralNetTools” R package.

### 2.5 Clinical correlation analysis

To investigate the relationship between SIGLECs expression and clinical features, we compare the expression of SIGLECs across six key clinical-pathological factors: age, stage, T-stage, M-stage, N-stage and gender. This comparison was conducted using COAD samples obtained from the TCGA-COAD dataset. The correlation analysis was performed using the Wilcoxon test (Krzywinski, 2014) for clinical features with 2 subgroups (age, gender and M-stage), but use Kruskal-Wallis test (Kruskal et al., 1952) for those with more than 2 subgroups (stage, N-stage, T-stage). The results were visualized using the “ggplot2” and “ggpubr” R packages.

### 2.6 Differentially expressed genes (DEGs) identification

Differential expression analyses were conducted using the “limma” R package (version 3.56) (Ritchie et al., 2015) (https://bioconductor.org/packages/release/bioc/html/limma.html). DEGs were identified based on a significance threshold, with a False Discovery Rate (FDR) < 0.05 and |log2FC| > 1 (Benjamini, 1995; Colaprico et al., 2016). The “ggplot2” R packages were used to visualize the DEGs results through the generation of volcano plots.

### 2.7 Protein-protein interaction (PPI) network

The overlapping DEGs were analyzed by STRING online databases (https://www.string-db.org/) to predict the PPI network and to determine the hub genes (confidence level 0.4) (Szklarczyk et al., 2015). The cytoHubba plugin of Cytoscape (Shannon et al., 2003; Li et al., 2022) was used to score each node gene by 10 randomly selected algorithms, including MNC (Maximum Neighbourhood Component), Degree, MCC (Maximal Clique Centrality), EcCentricity, EPC (Edge Percolated Component), Closeness, BottleNeck, Betweenness, Radiality, and Stress. The top 20 hub genes from each algorithm were used to screen hub genes through the “UpSetR” package.

### 2.8 Gene ontology (GO), kyoto encyclopaedia of genes and genome (KEGG)

To gain the possible roles of the genes within the significant module, we conducted analyses using GO and KEGG pathway. The GO terms are categorized into biological process (BP), cellular component (CC), and molecular function (MF). The “clusterProfiler” R package (version 4.8.2) was used for enrichment analysis and the “org.Hs.eg.db” R package (version 3.17.0) was used for ID conversion (Wu et al., 2021). Significance was established at different threshold, with a weak cut (*p*-value <0.05), an intermediate cut (*p*-value <0.01), and a strong cut (Benjamini-Hochberg adjusted *p*-value <0.05) (Benjamini et al., 2018) for GO terms, and an intermediate cut (*p*-value <0.05) and a strong cut (corrected q-value <0.05) for KEGG analysis.

### 2.9 Gene set enrichment analysis (GSEA)

The “clusterProfler” package (version 4.8.2) was used for GSEA analysis, which analyzes the relevant functions and pathways of genes. The results were visualized with “enrichplot” R package.

### 2.10 Immune infiltration analysis

The distribution of 22 immune cell types in both COAD and normal samples within the TCGA-COAD dataset was evaluated using the “CIBERSORT” algorithm (Newman et al., 2015; Chen et al., 2018). Subsequently, we calculated the correlations between SIGLECs and the immune cell composition using the “psych” R package. Additionally, we explored the associations between SIGLECs and four pivotal immune checkpoints—CD274, HAVCR2, LGALS9, and PDCP1LG2—using the “Spearman” correlation analysis (Fu et al., 2020).

### 2.11 Self-organizing maps (SOM) analysis

We apply the open-source tool “somoclu” (https://peterwittek.github.io/somoclu/) (Peter Wittek, 2017) to perform the SOM analysis. We reduce the 14-dimension SIGLECs data into 2-dimension for visualization, setting a Toroid-map with a resolution of 50*100. Based on the generated activation map, which quantifies the similarities between two different samples, we divide the full sample into different subsamples to study the evolution of COAD and the associated transition phases. Further correlation studies are also performed based on the SOM-defined subsamples.

### 2.12 Expression verification of SIGLECs

The immunohistochemistry results of SIGLECs in COAD were obtained by the Human Protein Atlas (HPA) database (Uhlén et al., 2015; Yao et al., 2022), accessible at https://www.proteinatlas.org/. This resource allowed us to explore the protein level expression and spatial distribution of SIGLECs in COAD and normal tissues.

## 3 Results

### 3.1 SIGLECs expression in different samples with PCA

**FIGURE 1:**
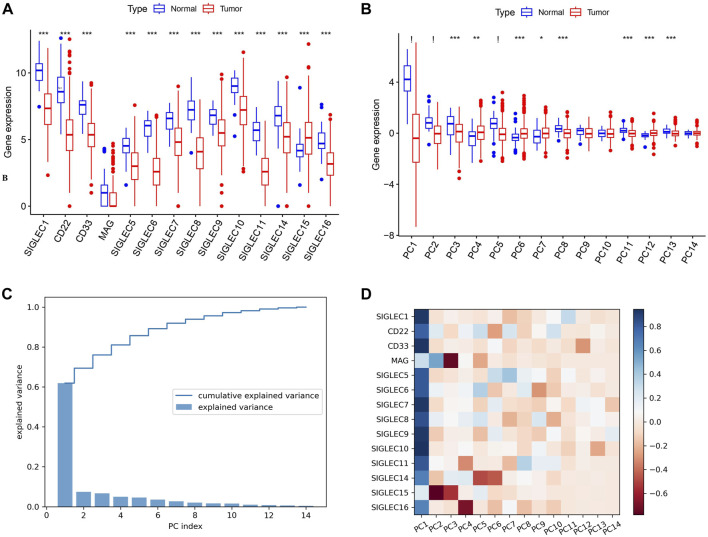
Expression of SIGLECs and its PCA performance. **(A)** This Boxplot depicts SIGLEC expression in normal tissues and COAD utilizing TCGA RNA-seq data. The significance level of each SIGLEC is shown on the top, with *** denoting *p* < 0.001. **(B)** The Boxplot for 14 PCs that correspond to 14 SIGLECs, with “!” indicates *p* < 0.000001. **(C)** The explained variance values for each PCs and their cumulative values. It indicates how the 14-dimensional SIGLECs expression information is distributed in different PCs. **(D)** The cross-correlation coefficient between each SIGLEC-PC pair, indicating the contribution from each SIGLEC in a chosen PC. Blue refers to a positive correlation, while red refers to a negative correlation.

Initially, we collect SIGLECs expression data from the TCGA database and compare the expression values of different SIGLECs in the normal/cancer cases, shown in Figure 1A. Our analysis revealed that only SIGLEC15 displayed a notably and significantly elevated expression, while 13 other SIGLECs (SIGLEC-1,5,6,7,8,9,10,11,14,16, CD33, CD22, and MAG) were consistently and significantly downregulated in COAD samples within the TCGA-COAD dataset (*p* < 0.001). To further understand this simultaneous change from normal cases to COAD patients, we use PCA to project the 14-dimensional SIGLECs data into 14 orthogonal principal components (PCs), i.e., PC1 to PC14. Similar to Figure 1A, their corresponding comparisons are shown in Figure 1B, but with their variations reducing from PC1 to PC14. We identify PC-1,2,5 as the hub PCs, with their statistical significances highlighted as “!” (*p* < 0.000001). We note the hub PCs are not treated as the hub genes, but as important roles in finding the hub genes during the COAD transition. The PCA results in Figure 1B suggest there are 11 out of 14 individual components that changes significantly in the normal-cancer transition, therefore, we choose to focus on the three most sensitive ones to add the COAD context for future analysis.

In Figure 1C, we show the SIGLECs’ information covered by each PCs in terms of the explained variance and its cumulative values. PC1 appears to occupy >60% of the total information/variance, while the top six PCs can explain >90% of the overall 14-dimensional information. By adding the contribution from all 14 PCs, 100% of the information of the 14 SIGLECs can be recovered. We demonstrate the detailed composition (from the SIGLECs) of each PC in Figure 1D, using the correlation coefficients. It is clear that PC1 is positively correlated with most of the SIGLECs, which can explain the simultaneous (normal-cancer) changes in Figure 1A. We further identify the strongest (anti-correlated) PC-SIGLEC pairs: PC2-SIGLEC15, PC3-MAG, PC4-SIGLEC16, PC5/PC6-SIGLEC14.

**FIGURE 2:**
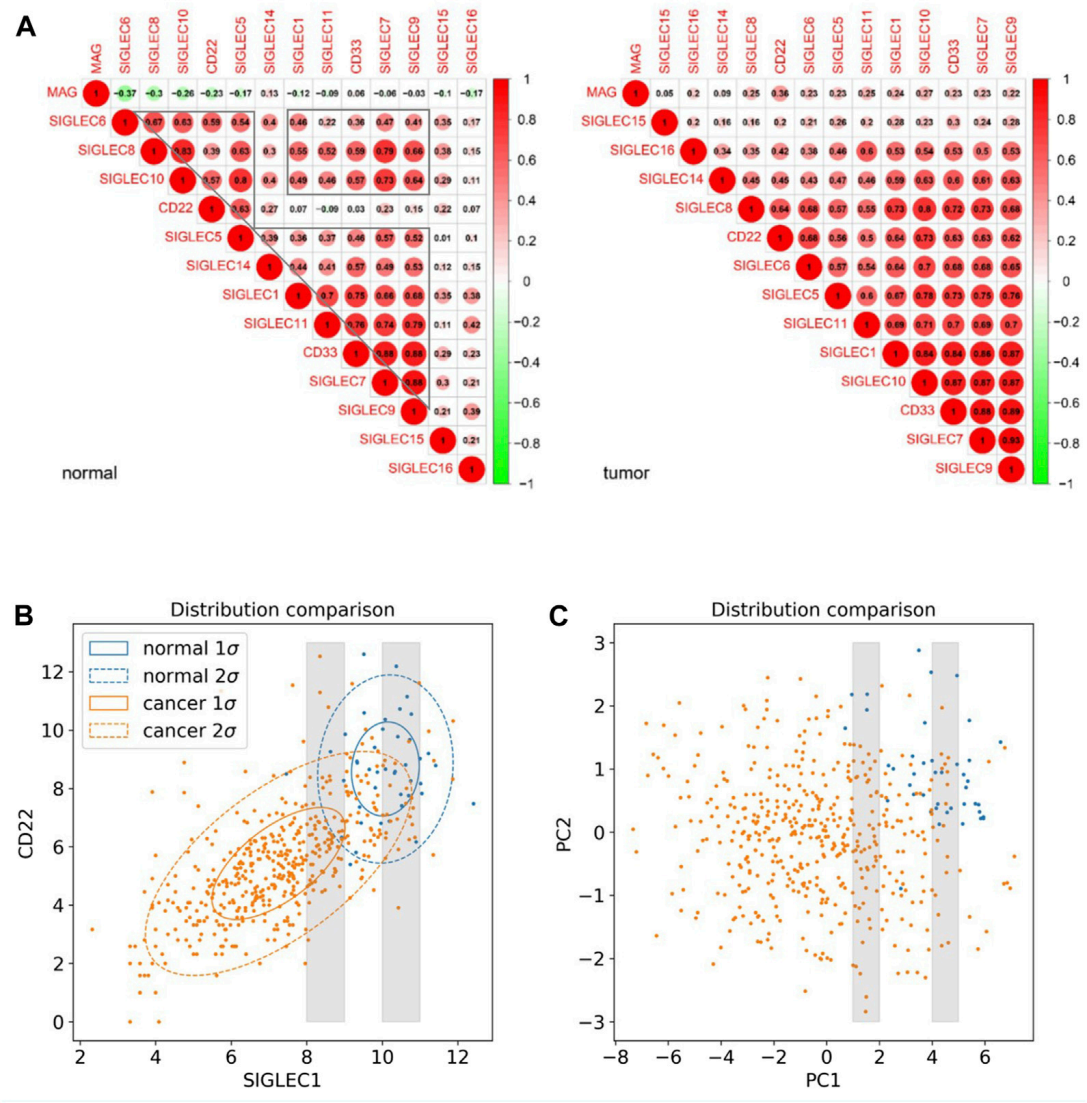
SIGLEC co-evolution in COAD. **(A)** Correlation coefficients for different SIGLECs in the normal sample (left) and COAD sample (right). **(B)** 2D scatter diagram of SIGLEC1 and CD22 to show the distribution change before and after getting COAD. **(C)** Similar to **(B)** but for PC1 and PC2.

Furthermore, we investigate the correlation, i.e., patterns of simultaneous change, between the 14 SIGLECs, and find most SIGLECs co-evolve after getting COAD. More specifically, we adopt the cross-correlation coefficient to quantify this change, shown in Figure 2A. It is shown that for the normal sample, the correlation coefficient values are clustered into three main areas, with their boundaries highlighted in grey in Figure 2A, while the values for the tumor sample are more concentrated in a single triangle. This is because we use a hierarchical clustering order for the SIGLECs, so that if one SIGLEC is more correlated to the others, they will be placed near each other in the figure. This pattern in the correlation coefficient suggest SIGLECs in the normal sample are co-evolving in two groups: SIGLEC-6,8,10, CD22, and SIGLEC5 are evolving together, while SIGLEC-5,14,1,11, CD33, and SIGLEC-7,9 are evolving together, leaving SIGLEC-15,16 and MAG almost independent from those two groups (later we will show the hub genes are from different groups). Most correlation coefficients also experience an significant increase from the normal sample to the tumor sample, suggesting COAD transition overwhelms some biological processes. Overall, it suggests COAD patients not only change SIGLEC expressions as shown in Figure 1A, but the correlations/connections between two different SIGLECs also increase. For example, we can see SIGLEC-9,7, and CD33 remain the strongest correlations with the others in both the normal sample and the tumor sample, while SIGLEC10’s correlation experienced a strong boost after getting COAD. Here we summarize the top seven most correlated SIGLECs in the tumor sample: SIGLEC-1,5,7,9,10,11 and CD33, meaning a single SIGLEC can represent a majority of another, so that they are less independent.

We use Figure 2B to further demonstrate the changes due to COAD. In this scatter diagram, each dot represents one person, with the expression of SIGLEC1 on the x-axis and the expression of CD22 on the y-axis. Having COAD will lead to a decrease in both SIGLECs’ expressions, so that the center of the orange distribution is at the lower-left corner compared with the blue distribution. This was demonstrated also in Figure 1B. But we also notice SIGLEC1 and CD22 become more correlated for COAD patients, as their distribution contour in orange is more elliptically distorted. How much more distorted the elliptical distribution is can be shown by comparing the SIGLEC1-CD22 value in Figure 2A: 0.07 for normal (which correspond to why they belong to different groups in the previous paragraph) and 0.64 for COAD (the elongated distribution making one SIGLEC associate to the other’s change, leading to less independence). This figure also demonstrates that exploring a single SIGLEC v.s. cancer feature might not be optimal. A method that can link multiple SIGLECs’ status, i.e., a multi-dimensional analysis is important. For instance, PC1 in Figure 1D can explain a majority of the correlated direction in different SIGLECs, thus it can represent the elongated direction in Figure 2B, while the other PCs can represent other directions in a full 14-dimensional scenario. We remark that as all the PCs are orthogonal to each other, their cross-correlation coefficient is zero, therefore we do not need to show their counterparts for Figure 2A.

Interestingly, PCA can also work as a tool to translate statistical correlation into causality in this highly correlated SIGLEC-COAD system. Generally, causal inference requires fine binning/sub-sampling or re-weighting to control the variables, and it is extremely hard to do so when the system is noisy and highly correlated. We use Figure 2B & 2C as examples: (1) If we want to control other variables, say SIGLEC1, and observe if CD22 is differently expressed for normal/cancer samples, we can select data within one of the grey narrow bins in Figure 2B. We can see that selections in SIGLEC1 will lead to selections in CD22, due to their strong correlation that makes them less independent. The noisy distribution will make it worse, so that we cannot significantly distinguish the normal/cancer samples. (2) After performing PCA, by definition, the system is transformed into orthogonal PCs, so that any selection in PC1 will not affect the distributions in PC2, as well as all the other PCs. Thus, in the grey narrow bins that controls PC1 in Figure 2C, we can still see a clear separation between normal/cancer samples in PC2. In this way, we confirm there exists a lot of independent components in the complicated SIGLEC family that are directly linked to COAD (Figure 1B).

### 3.2 COAD prognosis enhancement with PCA

**FIGURE 3:**
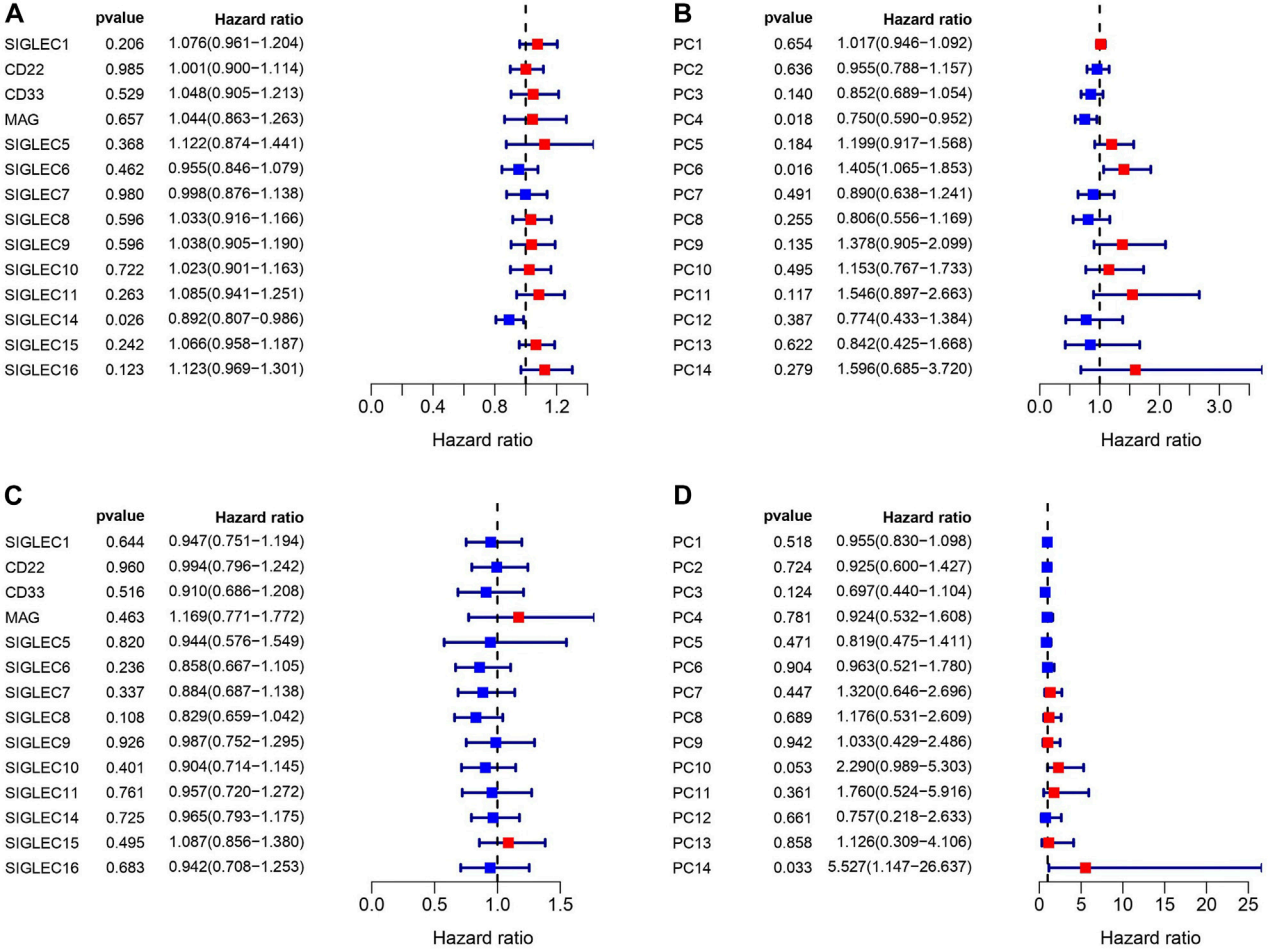
Correlations between SIGLECs/PCs expression and COAD patient survival. **(A)** Hazard ratio (HR) of SIGLECs for patients’ Overall Survival (OS). **(B)** HR of PCs for patients’ OS. **(C)** HR of SIGLECs for patients’ Disease Free Interval (DFI). **(D)** HR of PCs for patients’ DFI.

We match the SIGLECs expression data with COAD patients’ prognosis data, and separate the SIGLECs expression (or its PCs) into two groups with an equal number of people, to investigate how SIGLECs (and their associated PCs) affect patients’ survival rate as a function of time, i.e., the survival curve. In evaluating the prognostic significance of SIGLECs in COAD, distinctive associations emerged. In Figure 3A, we show the Hazard Ratio (HR) of the OS time, with SIGLEC14 being a significant (*p* = 0.026) positive indicator (HR < 1). The HRs for the corresponding PCs are shown in Figure 3B as a comparison. Similarly, in Figure 3C, we show the HR of the DFI time, however, without any significant (*p* < 0.05) indicators for prognosis. The HR of the DFI for the PCs are shown in Figure 3D.

**FIGURE 4:**
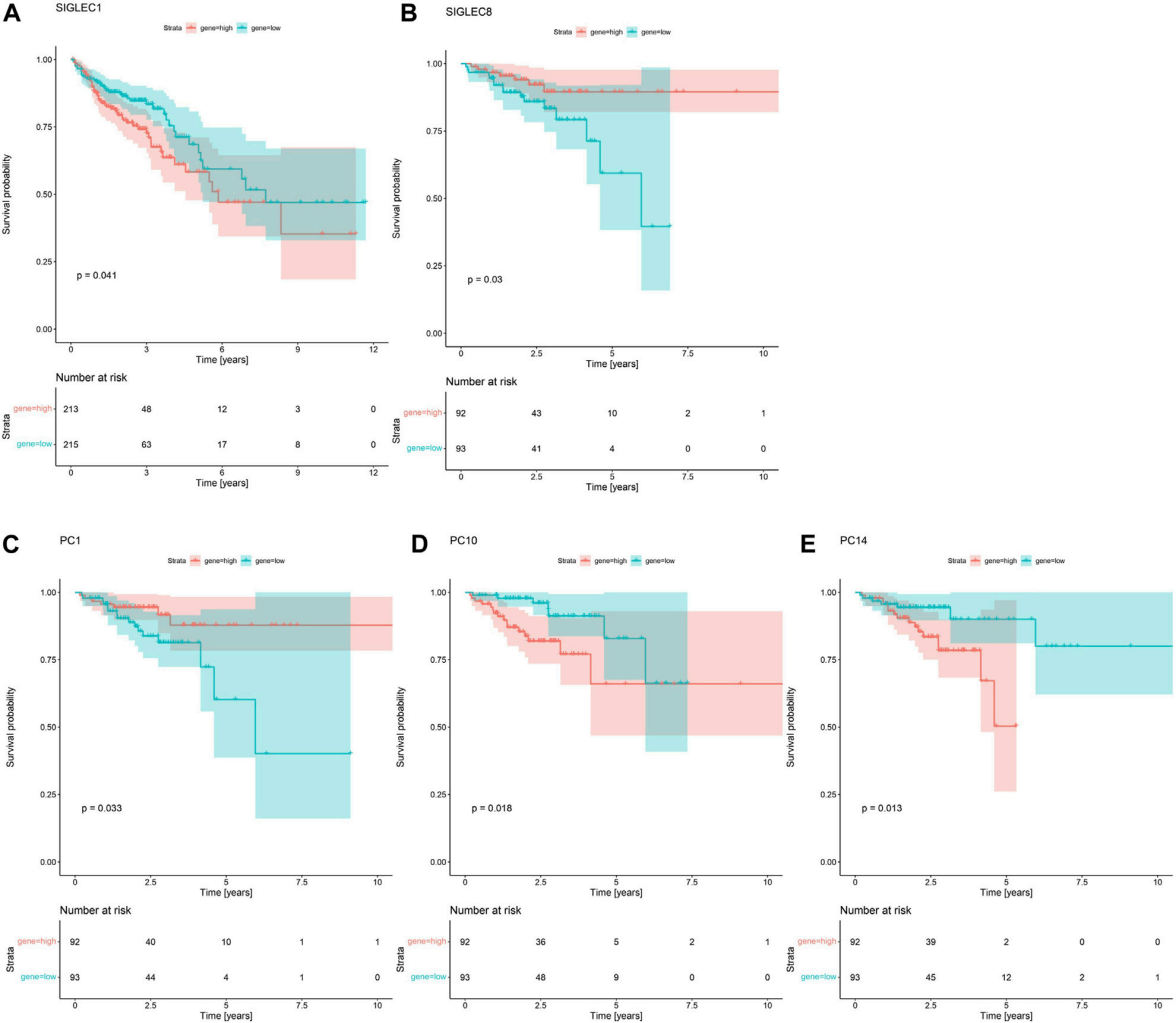
Survival Curves for SIGLECs and PCs. **(A)** Survival Curves for SIGLEC1 in association with patients’ OS in COAD. Red represents the high expression group, and cyan represents the low expression group. **(B)** Survival Curves for SIGLEC8 in association with patients’ DFI in COAD with the same color-coding. **(C)** Survival Curves for PC1 in association with patients’ DFI. **(D)** Survival Curves for PC10 in association with patients’ DFI. **(E)** Survival Curves for PC14 in association with patients’ DFI.

In Figure 4A, we demonstrate that SIGLEC1’s high expression can significantly (*p* = 0.041) suppress the survival probability considering the Overall Survival (OS) time. In Figure 4B, we show that SIGLEC8’s high expression can also significantly (*p* = 0.03) benefit the patient’s Disease-Free Interval (DFI) time. Therefore, SIGLECs can work as a potential indicator for COAD prognosis.

Interestingly, when comparing the power from the PCs, we find no PC can exceed the significance of SIGLEC1 considering the Survival Curve for OS time. This further emphasizes the importance of SIGLEC1. On the other hand, we find PC-1,10,14 are very competitive indicators considering the DFI time, compared with SIGLEC8 in Figure 4B. They have significant impacts on DFI time with *p* = 0.033 (Figure 4C), *p* = 0.018 (Figure 4D), and *p* = 0.013 (Figure 4E). It suggests PCs are even better tracers for prognosis, especially considering they are orthogonal to each other, so a combination of different PCs can give a much clearer result.

**FIGURE 5:**
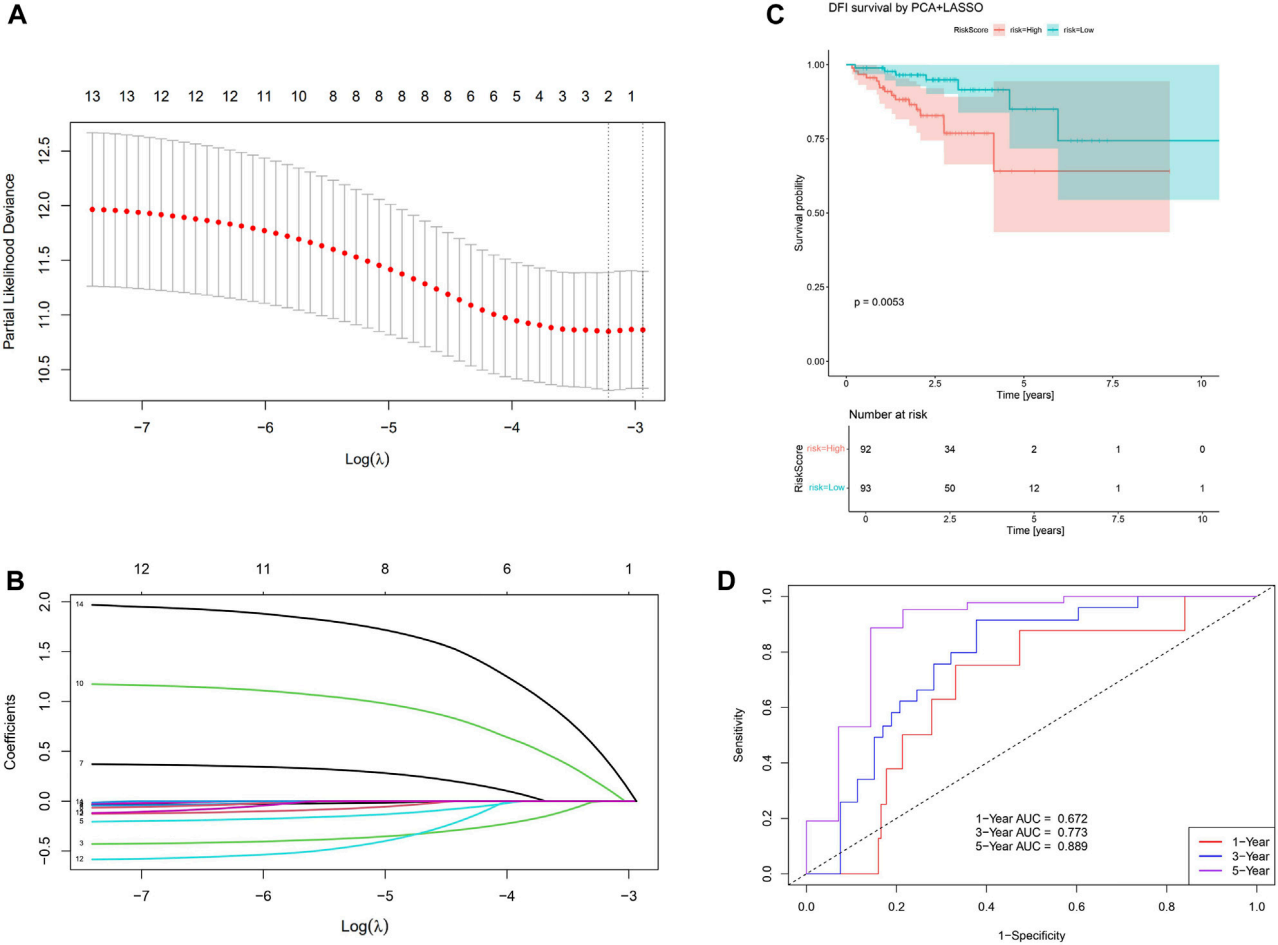
Results for the PCA+LASSO+multivariate Cox analysis. **(A)** Finding the value for the tuning parameter λ . **(B)** Decide the coefficient given λ . **(C)** Use the selected PC-10,14 to build a multivariate Cox model and the corresponding survival curves. **(D)** AUC for the model at 1,3,5 years.

To further extend the capability of PCA, we perform a multivariate Cox regression together with PCA and LASSO algorithms. With LASSO, we confirm PC-10,14 affect the resulting risk at significant level (Figures 5A, B), therefore they be treated as two inputs for the Cox regression. With the multivariate Cox analysis, we separate the sample into high/low risk subsamples using the resulting risk evaluation. We find they are significantly (*p* = 0.0053) separated considering the DFI time survival curve, as shown in Figure 5C. The corresponding one/three/five years area-under-curve (AUC, Figure 5D) also suggest using two PCs already give very promising predictions.

We find that SIGLEC15 manifested as a negative factor (HR > 1), whereas SIGLEC-6,7,14 emerged as positive factors (HR < 1) in both DFI and OS analyses for COAD (Figures 3A, C). Unfortunately, none of their significances exceed the *p* < 0.05 limit, possibly due to the limit of the current data size. With PCA, the significance can be largely improved. In the OS analysis, PC-4,6 exhibit a strong deviation from HR = 1, while in the DFI analysis, PC-10,14 also offer strong evidences. Nevertheless, PC-10,14 also shows strong differences in Survival Curves as shown in Figures 4D, E. All the above results in Figures 3–5 suggest PCA can dramatically improve the significance of using SIGLECs in COAD prognosis. These findings underscore the potential of SIGLECs, especially their PCs, to serve as prognostic markers, providing valuable insights into the clinical outcomes of COAD patients.

### 3.3 COAD prognosis sensitivity and full SIGLEC information application with NN

As demonstrated previously, single SIGLEC v.s. prognosis sensitivity is not optimal currently with TCGA data, possibly due to the noisy data with very limited sample size. Even with PCA enhancement, we can merely use two PCs to perform the multivariate analysis. So, the current data quality does not support us for a full-parameter-space, full-information analysis. We only use NN to show its capability in future prognosis analysis.

**FIGURE 6:**
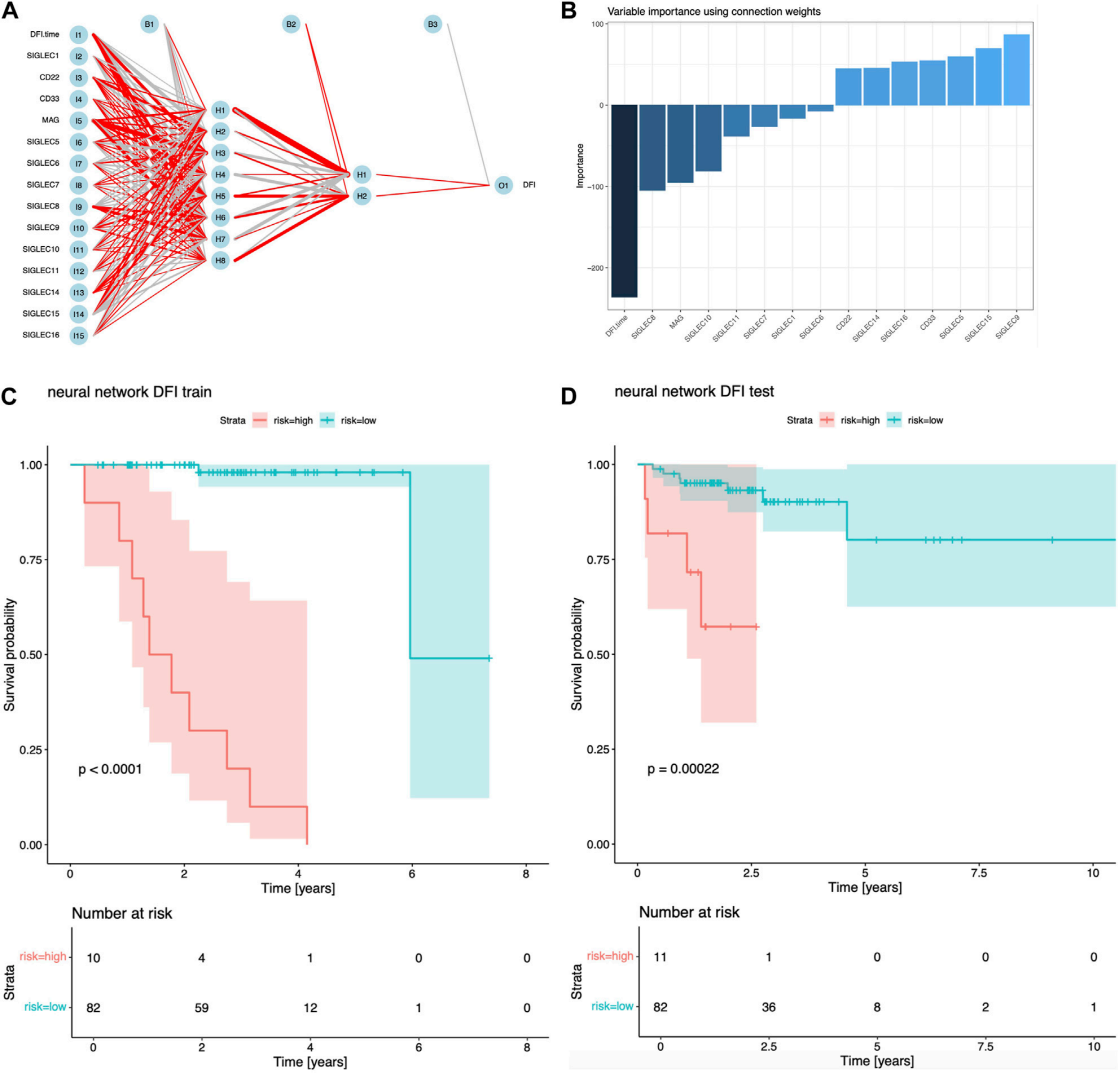
Results for NN analyisi. **(A)** Visualization of the structure and trained hyper-parameters of the network. **(B)** Importance of the input features from the products of the connection weights. **(C)** Survival curves using the NN predicted risks for the training sample. **(D)** Similar to **(C)**, but for the test sample.

In Figure 6A, we show the structure of the NN, with positive/negative contributions colored in red/grey, while thick/thin lines denote large/small contributions. The sensitivity of risk v.s. individual feature can be visualized by the partial derivatives or the products of the connection weights (Olden et al., 2004), which we show in Figure 6B. The DFI time dominates over all the SIGLECs, which we do expect, as the relative measurement noise for time is clearly significantly smaller than in the gene expressions, making it easier to subtract information from the DFI time for the NN. We find SIGLEC9 dominates the positive contribution, while SIGLEC8 dominates the negative contribution. Most SIGLEC sensitivity do not differ too much, due to the complicated biological correlations as well as similar signal-to-noise level.

We find NN is a good sanity check for PCA, as SIGLEC9 is one of the main (and the only positive) contributors to PC14, and SIGLEC 8 is the most significant contributor to PC10, which PC-10,14 are the most important PCs that affect DFI survival according to Figures 1D, 4, 5. This confirmation highlight the good linearity in the data, and suggests NN is not strongly biased by contaminations.

We show the performance of the training sample and the test sample in Figures 6C, D. It is clear both the training sample and the test sample can have a significant separation of high/low risk subsamples. The test sample exhibit a stronger significance (*p* = 0.00022), which is stronger than the previous combination of PCA, LASSO, and Cox regression. But we note this is not a fair comparison, as the previous analysis uses only two PCs, while this NN approach uses all 14 SIGLECs, containing all the information from 14 PCs, plus possible non-linear/non-Gaussian information that PCA ignores, plus (most importantly) a much stronger sensitivity from DFI time measurement.

This NN approach is only for the demonstration of its great potential in the future. We will address its limitations in the Discussion section.

### 3.4 Clinical correlation analysis enhancement with PCA

**FIGURE 7:**
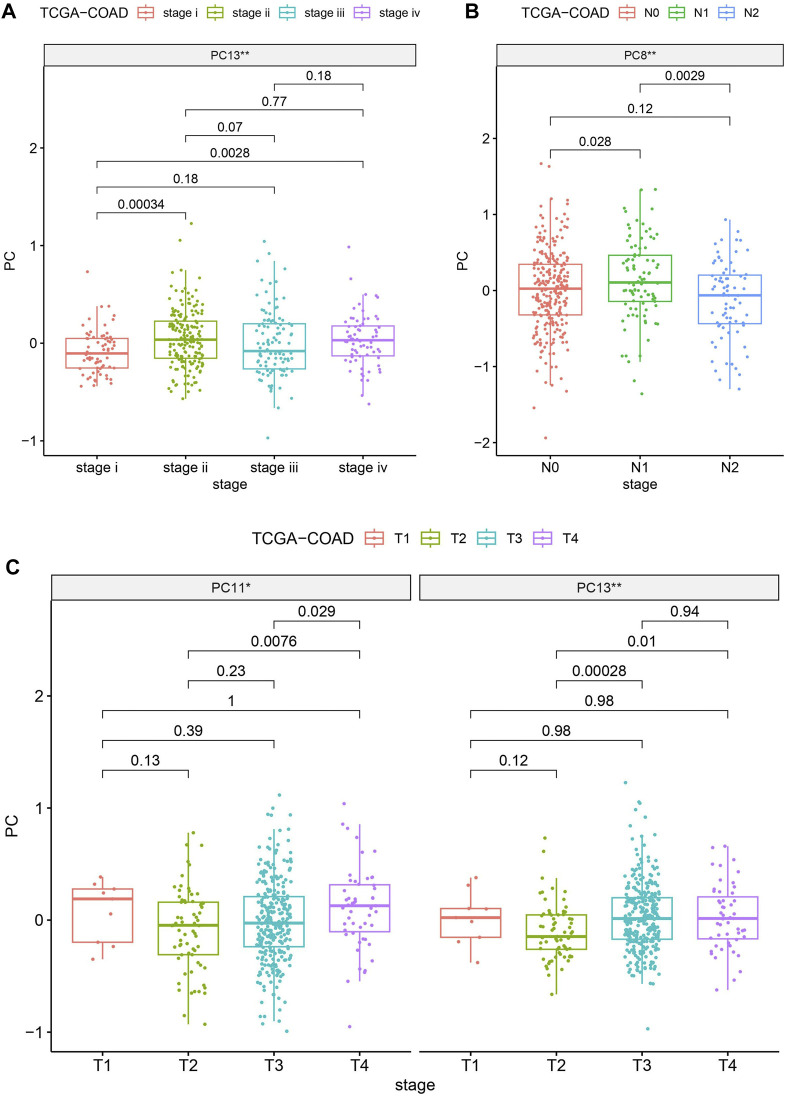
Evaluating the significances (** for *p* < 0.01) of SIGLECs (or PCs) in COAD with different clinical features. **(A)** PC v.s. Stage correlation; **(B)** PC v.s. N-stage correlation; **(C)** PC v.s. T-stage correlation.

To gauge the potential significance of SIGLECs in the progression of COAD, we examine the correlation between SIGLECs (or PCs) and key clinical features. We specifically focused on Age, Gender, Stage, Metastasis (M), Lymph Node Involvement (N), and Tumor Size (T). We find almost null detection (see Supplementary Figure S6) across all these clinical features for the SIGLECs, however, PCA can find significantly correlation in terms of Stage, N-stage and T-stage, as shown in Figure 7. Figure 7A exhibits PC13 has a much stronger correlation with COAD stage than any individual SIGLEC. Similarly, in Figure 7B, PC8 shows a significant correlation with COAD N-stage. Last but not least, Figure 7C highlights the significant correlation between PC13 and COAD T-stage. The above analysis further confirms the importance of using PCA in the highly correlated multi-dimensional SIGLECs data, so that its correlation with different definitions of COAD stages is revealed. Interestingly, Figure 7 reveals a non-monotonic correlation between PC13 and COAD stage and T-stage, which suggest PC13 should be involved in complicated biological processes that associate with COAD in multiple but opposite ways.

### 3.5 DEGs, PPI and enrichment analysis of COAD

**FIGURE 8:**
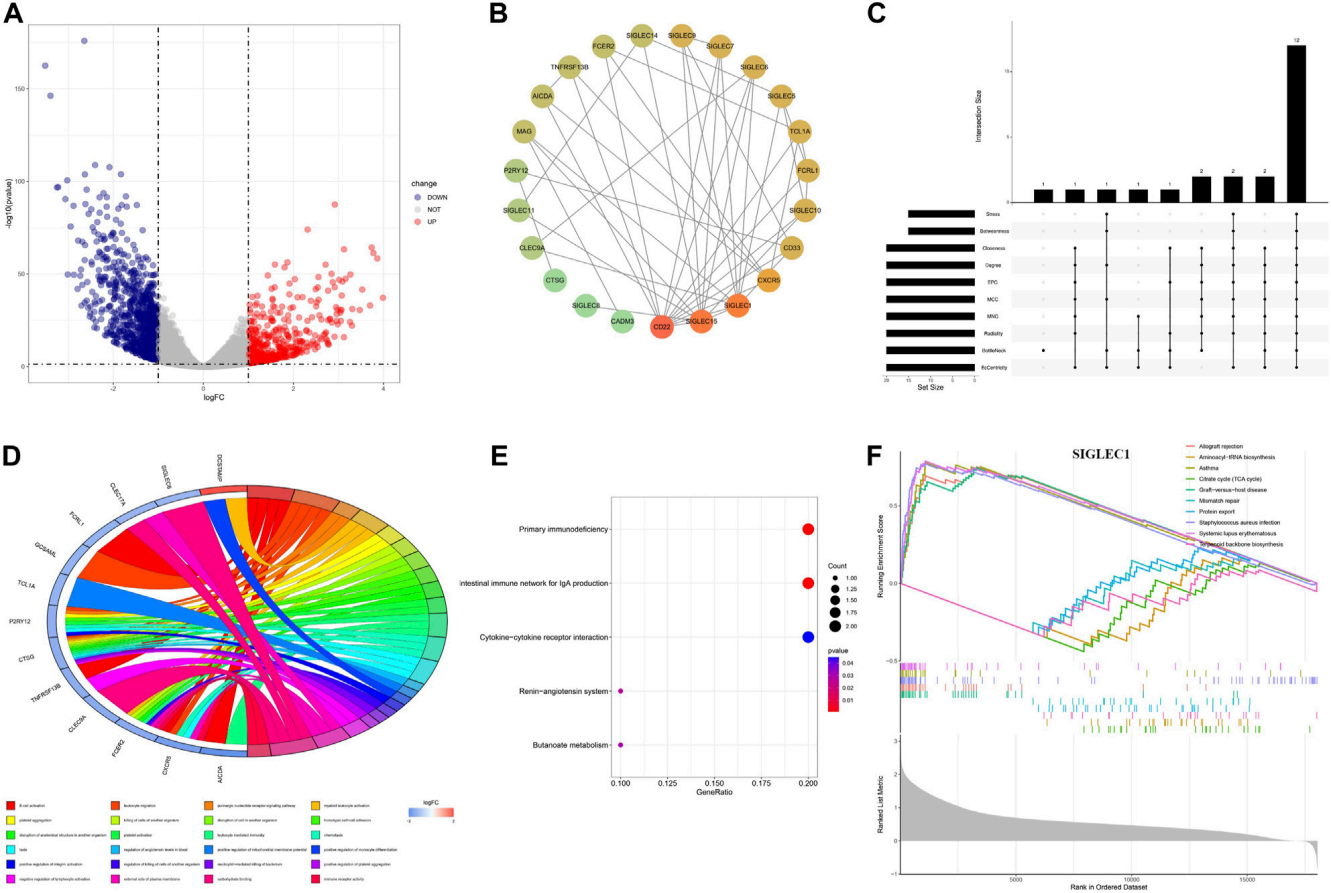
Differential expression analysis and the corresponding enrichment analysis. **(A)** Volcanic plots depicting differentially expressed genes in both normal and cancer groups are presented. **(B)** PPI network of SIGLECs and the selected DEGs. **(C)** Venn diagram showing genes associated with SIGLEC expression and differentially expressed in COAD. **(D)** Circular diagram illustrating enriched GO entries. **(E)** Bubble plot of KEGG pathway enrichment analyses. **(F)** Single-gene GSEA enrichment results for SIGLEC1.

We identified the differentially expressed genes (DEGs) to unravel the co-evolution of other genes together with SIGLECs in COAD. We find 1,254 DEGs, comprising 449 upregulated and 805 downregulated genes, shown in Figure 8A. By intersecting the 1,254 DEGs with 949 genes that demonstrated high correlations with SIGLECs, a set of 18 overlapped key genes emerged. We analyzed these DEGs and construct a PPI network with medium confidence (score >0.4), depicted in Figure 8B. The rank of genes was demonstrated by color changes, and the first ranked gene was marked with red bubble. We assess the significance of each node gene through 10 randomly selected algorithms, including MCC, MNC, EPC, Degree, BottleNeck, Closeness, EcCentricity, Radiality, Betweenness, and Stress. The top 20 hub genes identified by each algorithm were then compared, and the common hub genes across all 10 algorithms were determined, as illustrated in Figure 8C. Ultimately, we identified 12 overlapping hub genes (CD22, SIGLEC15, SIGLEC1, SIGLEC6, SIGLEC10, CD33, CXCR5, SIGLEC9, SIGLEC5, FCRL1, TCL1A, and MAG).

The DEGs are involved in various biological processes, including immune regulation, cell-cell interactions, signaling, etc. This finding is consistent with literature (Lubbers et al., 2018). In terms of functionality, these 18 key genes exhibited significant enrichment in processes such as B cell activation, leukocyte migration, platelet aggregation, and more, encompassing a total of 216 Gene Ontology (GO) functions that suit the *p* < 0.05 cut. Among those GO terms, a more cautious cut of *p* < 0.01 highlights 24 of them (Figure 8D). Under Benjamini-Hochberg correction, the corrected *p* < 0.05 highlight only two of them: B cell activation and carbohydrate binding. Additionally, the Kyoto Encyclopedia of Genes and Genomes (KEGG) pathways associated with these key genes included primary immunodeficiency, intestinal immune network for IgA production, renin-angiotensin system, cytokine-cytokine receptor interaction, and butanoate metabolism (Figure 8E), while the q < 0.05 cut highlight the first two.

We conducted Gene Set Enrichment Analysis (GSEA) to uncover relevant signaling pathways and potential biological mechanisms associated with SIGLECs. The x-axis in the GSEA represents individual genes, with each small vertical bar symbolizing a specific gene. The overall trend of the pathway, whether it is trending upward or downward, provides insights into the potential activation or inhibition of specific pathways. For each gene, the GSEA reveals the top five entries that are upregulated and downregulated (Figure 8F). As illustrated in the figure below using SIGLEC-1 as an example, this GSEA analysis serves as a valuable tool for identifying key pathways and unraveling potential biological mechanisms associated with SIGLECs in COAD.

### 3.6 Association of SIGLEC expressions with immunotherapy

**FIGURE 9:**
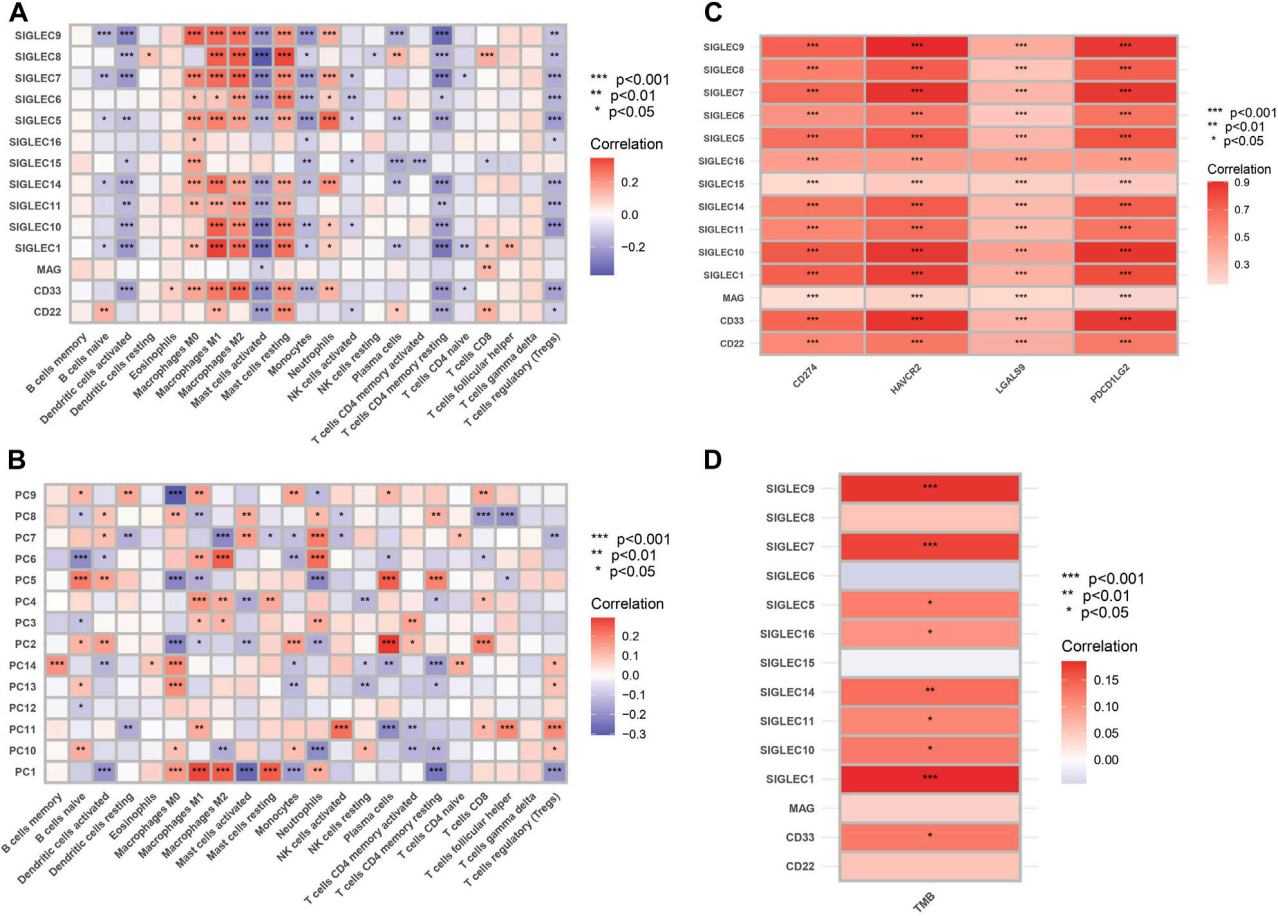
**(A)** Heat map of correlation between SIGLECs and immune cells. The color indicates the correlation strength, with red signifying positive correlations and blue indicating negative correlations. **(B)** Heat map of correlation between PCs and immune cells. **(C)** Correlation between SIGLECs and immune checkpoints. **(D)** Correlation between SIGLECs and TMB.

We examine the correlation between SIGLEC gene expression and 22 immune cell content, shown in Figure 9A. The findings revealed a significant negative correlation between genes related to T cells CD4 memory resting, activated Mast cells, and Dendritic cells when compared to other immune cell types. The activation of SIGLECs may be associated with immune evasion, leading to a decreased aggressive immune response against tumors (Macauley et al., 2014). Conversely, Macrophages M1, Macrophages M2, and various other immune cells exhibited marked positive correlations. There may exist an immuno-modulatory mechanism connecting SIGLECs activation and the inflammatory response of macrophages. These findings provide valuable insights into the potential impact of SIGLEC expressions on the immune cell landscape. We will revisit this point later with the SOM machine-learning algorithm.

To further understand the complicated correlations between SIGLECs and immune cells, we nonetheless apply the PCA and show the correlation between the corresponding PCs and immune cells in Figure 9B. We see that with PCA, the correlated patterns appear to be more clear: (1) some null or weak correlations between SIGLECs and immune cells start to reveal their core and significant hidden truth with PCA, i.e., in B cells memory, Dendritic cells resting, NK cells activated, T cells CD4 naive, T cells follicular helper, etc.; (2) some immune cells with strong correlations to most SIGLECs appear to be due to correlations to limited PCs, i.e., Macrophages M0, M1, M2, Mast cells resting, T cells CD4 memory resting, etc.; (3) PC-1,5 appear to be strongly correlated with most immune cells, which confirms the findings in Figure 1B. This again demonstrated PCA is a powerful tool that can subtract the key features as well as enhance the signal strength.

We show significant positive correlation between SIGLECs and immune checkpoints, including CD274, HAVCR2, LGALS9, and PDCD1LG2, in Figure 9C. The analysis of Tumor Mutational Burden (TMB) in relation to SIGLECs demonstrated significant positive correlations, as shown in Figure 9D. SIGLEC-1,7,9,14, exhibited notable positive correlations with TMB. This provides a detailed understanding of how SIGLECs influence the immune environment.

### 3.7 A multi-dimensional analysis of the SIGLECs with SOM

**FIGURE 10:**
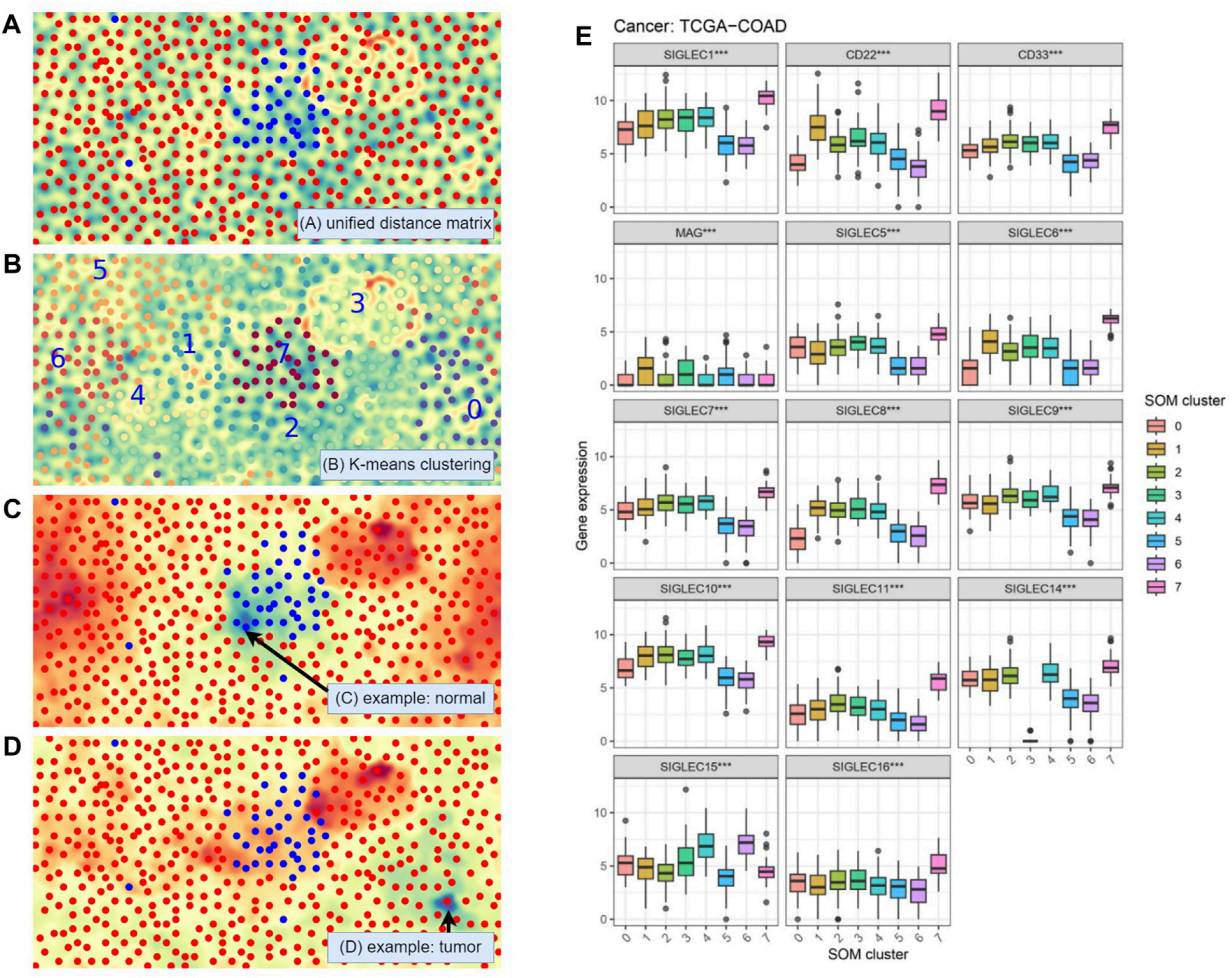
Self-Organizing Maps (SOM) unsupervised learning analysis. **(A)** Visualization with the Unified Distance Matrix (U-matrix) of SOM, demonstrating SIGLECs are sensitive to COAD cancer cells. **(B)** Automatic categorization with SOM and K-means algorithm, divided into 10 sub-samples. **(C)** Visualization of the activation map of SOM, to show the similarities (cold color: similar, warm color: different) to a chosen normal reference. **(D)** The activation map when choosing a COAD patient as the reference. **(E)** SIGLECs expressions that strongly evolve with different SOM subsamples.

As discussed above, the connection between SIGLECs and COAD should be revisited at higher dimensions considering the complex correlation between different SIGLECs (Figure 2). We therefore adopt the SOM algorithm, which was historically developed to reduce dimensionality, visualization, and pattern recognition (Kohonen, 1982). We reduce the 14D SIGLECs’ expression data onto 2D and show the corresponding unified distance matrix (U-matrix, which specifies the distance/similarity between a specific pixel and the neighboring pixel) in Figure 10A. The normal samples (blue) are clustering in the center, giving only SIGLECs’ expression data fed to the SOM algorithm, among all the COAD samples (red). The background U-matrix color-map also exhibits that the normal samples are similar to each other, with a blue-green color showing each dot/pixel is similar to its neighbors. We note there are three normal cases mixed into the COAD cases, possibly due to noise in the data (measurement/instrument error, individual difference, or contamination from other diseases, etc.)

In Figure 10B we apply a K-means clustering algorithm on top of the SOM map being generated. The full sample is divided into eight subsamples, and we arbitrarily assign a number from 0 to 7 to them, while each sub-sample is marked with a different color. Again, we see the machine learning algorithm defined a subsample “7” that is highly overlapped with the given normal sample, demonstrating the combination of 14 SIG-LECs can clearly identify the difference between normal and COAD samples. It is natural to think that the other seven subsamples could have other physical/medical meanings.

We use Figures 10C, D to show in the eyes of the SOM algorithm how the cases distinguish from one another. Figure 10C is the activation map that presents how different each case is from a chosen person (arrowed). The blue-green color denotes high similarities, while the red-orange color denotes large differences. We can see the chosen person is similar to the type-7 (normal) sample, while quite different from most of the other (cancer) samples. On the other hand, in Figure 10D we find this given patient is not only different from the healthy type-7, but also significantly different from type-3. This suggests except for identifying if a person has cancer cells, SIGLECs could potentially respond to other behaviors of cancer cells.

We further show the detailed SIGLECs’ expressions in those eight AI-assigned types in Figure 10E. In each individual SIGLEC, we find ultra-significant patterns of differences, rejecting the no-evolution hypothesis at $p<10^{-10}$ level with Kruskal-Wallis test. We choose the following case-study:

(1) SOM type-7: In general, type-7 are expressed higher than the other types, which is consistent with the healthy case in Figure 1A.
(2) SOM type-3: It contains 26 COAD patients, with most of the SIGLECs expressed similar to the other COAD types. However, its SIGLEC14 expression is extremely low, at almost 0. Later we will show type-3 have other effects, thus this is not likely due to bad data.
(3) SOM type-0: It is significantly under-expressed in CD22 and SIGLEC-6,8,10.
(4) SOM type-5,6: These two types have interesting similarities of low expressions in SIGLEC-1,5,6,7,8,9,10,11,14, CD22 and CD33, however, they differ a lot in SIGLEC15.

**FIGURE 11:**
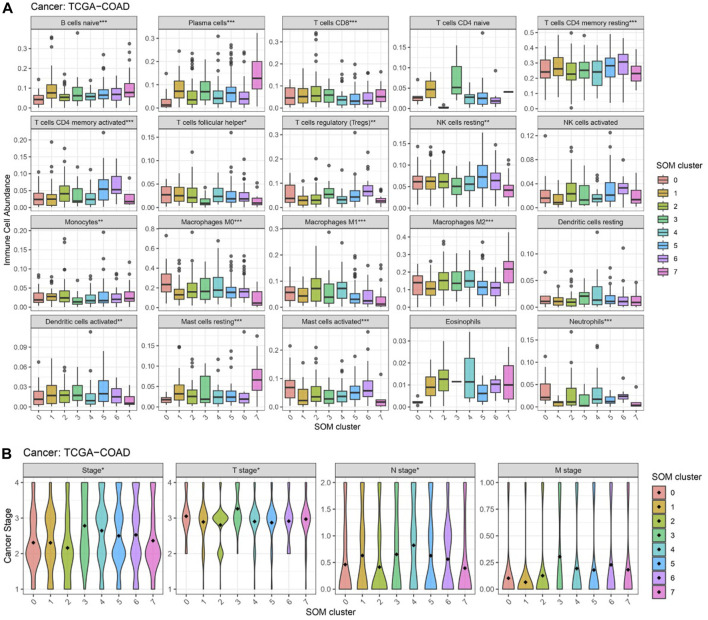
Changes in other features that correspond to different SOM subgroups. **(A)** Immune cell abundances are different, especially for type-7,3,0,5&6 **(B)** Cancer stages are different, especially for type-3.

We note the SOM classification is only based on the SIGLECs expression levels, which could potentially suffer from measurement bias, equipment bias, or inter-individual variations. Thus, to tell if the separated subsamples are due to true evolutional differences or noise, it is essential to check whether the other biological features change or not. In Figure 11, we present the validations of the SOM subsamples with immune cell abundance and cancer stages. In Figure 11A with immune cell abundance, we find:

(1) SOM type-7 is significantly different in Plasma cells, Macrophages M0, Macrophages M2, and Mast cell resting. This suggests these four immune cells could play the most important role during the healthy-cancer transition. Others such as NK cells resting, Macrophages M1, and Mast cells activated also have a similar behavior, but less significant.
(2) SOM type-3 is significantly under-expressed in T cells follicular helper and Neutrophils, while the significance is lower in Monocytes. Nevertheless, we see type-3 also represents the latest Stage and T-stage in COAD, as shown in Figure 11B. This indicates this type could also be physical, and its very low SIGLEC14 might not due to measurement errors.
(3) SOM type-0 is significantly under-expressed in B cells naive, Plasma cells, Eosinophils, and over-expressed in Macrophages M0, Mast cells activated. The fact that immune cells co-evolve with SIGLECs also suggests this type is realistic.
(4) SOM type-5,6 also have similar behaviors in immune cells, similar to their SIGLECs. They are highly expressed in T cells CD4 memory activated, NK cells resting, confirming the similarities in Figure 10E.

From the above results, it is clear SOM can further separate the COAD patients into subgroups with features that change in both SIGLECs and other properties such as immune cells or cancer stages. These subgroups correspond to potential COAD cancer subtypes, possibly with different origins or with different transition phases. Additionally, as these potential subtypes are identified only with SIGLECs expression data, it is possible to extend SIGLECs for future treatments.

We, therefore, argue the SOM categorization is a very promising tool to unveil COAD developments and its associated origin or transition phases with SIGLECs. We further note that due to some missing data in the original database, we choose to remove B cells memory and T cells gamma delta in Figure 11A, as some values have no measurement or zero standard deviation.

### 3.8 Cancer pathway activation and drug resistance

**FIGURE 12:**
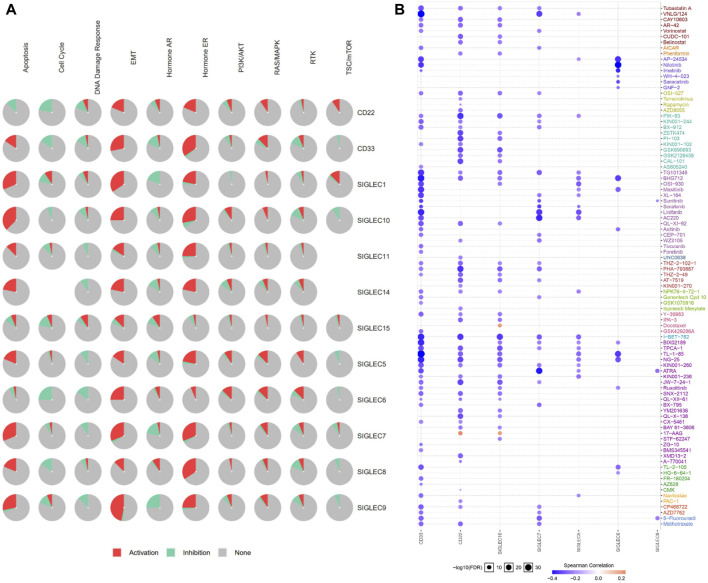
**(A)** SIGLECs cancer pathway activation. **(B)** Relationship between SIGLECs expression and drug resistance.

We utilized the GSCALite to examine the participation of SIGLECs in 10 common cancer pathways. The analysis showed that almost all of SIGLECs are involved in activating Hormone ERE and MT pathways. (Figure 12A). This insight clarifies the potential impact of SIGLECs on key cancer-related pathways.

In treating COAD, it is crucial to evaluate patient drug responses. To achieve this, the correlation between drug susceptibility and SIGLECs expression was investigated based on data from the Cancer Drug Susceptibility Genomics (GDSC) database. The analysis encompassed a diverse list of drugs. The correlation analysis resulted in the identification of seven SIGLEC-drug relationships (Figure 12B). Significantly, CD33 showed the highest correlation with other drugs, possibly due to the current extensive research focus on drug treatments related to CD33 (Linenberger, 2004).

### 3.9 SIGLECs expression level verification

During the validation of SIGLECs expression levels, an analysis was conducted using the GSE110224 dataset. The results generally agree with our finding in Figure 1, with slight differences in SIGLEC-10, which is due to the limited data size of this test sample (Supplementary Figure S8). More importantly, we note the 34 samples from GSE110224 are obtained from 17 patients, with one COAD sample and one normal sample from each patient. This largely rule out the possible contamination from interindividual variability. During the validation with a larger dataset GSE39582, we search for very significantly expressed (***, with *p* < 0.001) genes, but find only SIGLEC-1,6,16 (Supplementary Figure S8) in both TCGA and GSE39582. The fact they are all down-regulating in COAD confirms our findings. However, we note there are a few SIGLECs with inconsistent expressions in these two databases, but with low significance in at least one database. We think this is due to data imbalance, especially the lack of normal control samples in GSE39582.

**FIGURE 13:**
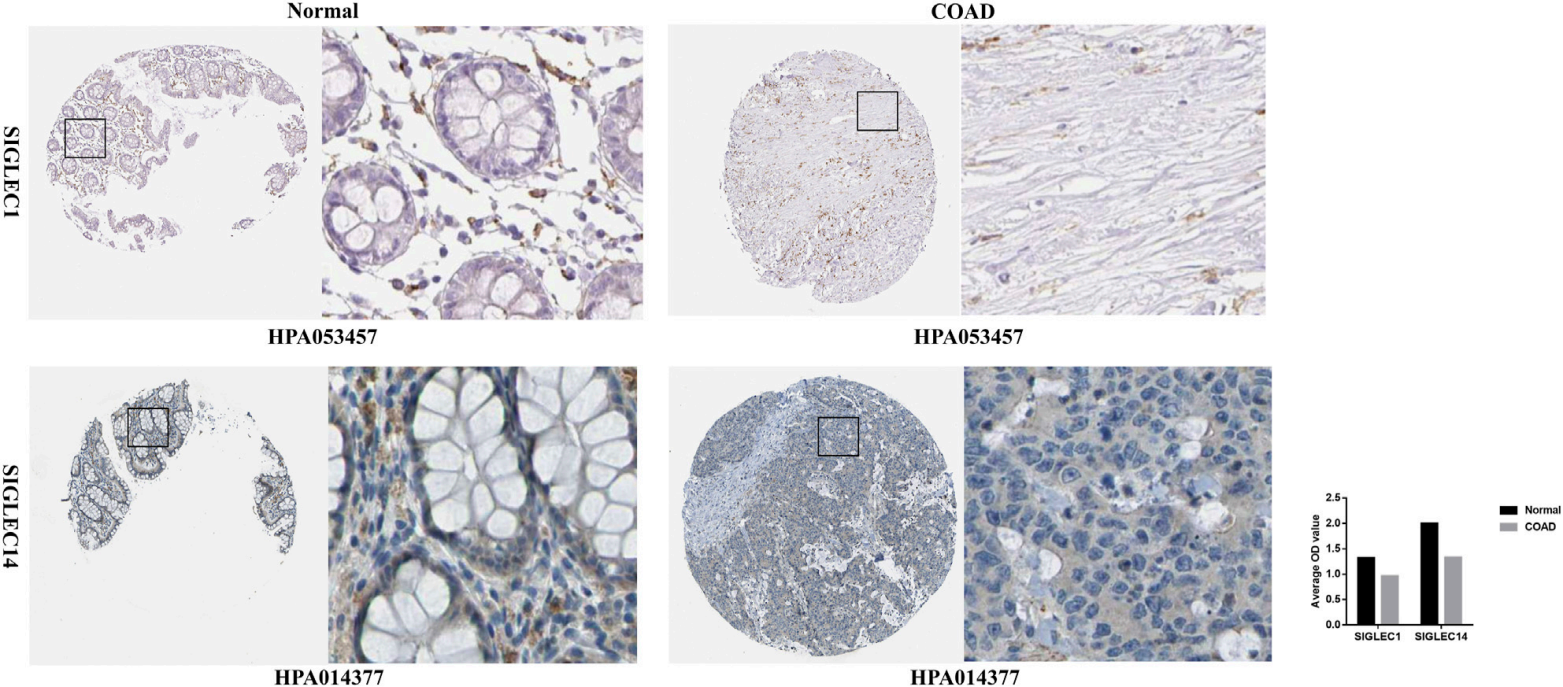
Immunohistochemistry of SIGLEC1 and SIGLEC14 in COAD and normal tissues from the Human Protein Atlas database.

Furthermore, for a deeper investigation into SIGLECs’ protein expression levels, we acquired immunohistochemistry data for SIGLECs in both COAD tissues and normal tissues from the HPA database. Specifically, HPA053457 and HPA014377 were subjected to quantification using ImageJ. As depicted in Figure 13, the results revealed that SIGLEC1 and SIGLEC14 exhibited lower expression levels in COAD tissues compared to normal tissues.

## 4 Discussion

In this work, we perform a comprehensive study on multi-dimensional expressions of the SIGLECs, especially on their relations with COAD patients. Firstly, we find simultaneous suppression in most SIGLECs in COAD patients, while the PCA algorithm can subtract the most dominant component, i.e., PC-1,2,5. The PCA can also tell the projectional/fractional contribution of different SIGLECs in each PC, as demonstrated in Figure 1. We also find PC1 is highly correlated with all the SIGLECs which are significantly lower expressed in COAD patients, therefore it represents the key correlated feature of the SIGLECs (possibly corresponding to the elongated direction in Figure 2B). Interestingly, the elongated direction in Figure 2B is along the direction of the centers of normal/cancer distributions, confirming the linearity assumption that validates the application of PCA. Secondly, we investigate the SIGLEC-SIGLEC cross-correlation coefficient in normal/cancer cases, and find significantly enhanced correlations in the COAD patients, as shown in Figure 2.

We present the HR calculations in Figure 3. We see that SIGLEC14 is the only significant (*p* < 0.05) tracer in the HR analysis considering the OS time, but with PCA, we find PC-4,6 are both strong tracers. Similarly, considering the DFI time, we find no significant tracer from the SIGLECs, but PC-10,14 from the PCA. In Figure 4, we present the Survival Curves, showing that low SIGLEC1 can significantly benefit the OS time, and high SIGLEC8 can significantly benefit the DFI time. This highlights the potential of using SIGLECs in prognosis studies. More importantly, we find that PCA can significantly enhance the outcomes of DFI analysis, with three orthogonal/independent PCs strongly correlated with the survival rate, i.e., PC-1,10,14. The resulting significance can reach *p* = 0.0053 when a multivariate analysis is applied (only with PC-10,14), by combining PCA, LASSO and Cox regression, as shown in Figure 5. This further proves PCA can de-noise this highly correlated SIGLECs system and subtract the key biological information: the SIGLEC-survival correlation was not significant enough to support a LASSO+multivariate Cox regression, but PCA enables such analysis to happen.

We additionally validate our binning methods in the SI, as a sanity check. We follow the recommendations (Raman et al., 2019) and select quantile splitting and K-means clustering, as we think other methods like distribution based splitting, Cox regression model and D-index have their own assumptions on the data, while Figure 2B do not suggest strong deviations from Gaussian distributions. We extend the SIGLEC1 v.s. OS time and the SIGLEC8 v.s. DFI time survival analysis in Supplementary Figure S5, while also perform K-means clustering and splitting by <25% and >75%. We find very consistent results comparing with the fiducial median-binning. We note although K-means gives more significant separations, it is based on the unsupervised clustering in the time v.s. gene expression space with different units, while changing the units being used can significantly change the results. The splitting by <25% and >75% method will remove half of the available data, leading to a larger noise. Therefore, we stick to the binning by median method throughout this paper.

Alternatively, a NN is constructed to explore the potential of the SIGLEC family in COAD prognosis. Different from the above approach, in the NN, we choose to include all the SIGLEC expressions, despite how significantly they are correlated to the prognosis. In addition, the DFI time is also included as an input feature, which exhibit a stronger sensitivity than the SIGLECs in Figure 5B. We note NN is extremely powerful when given a larger and unbiased dataset, and is therefore very limited now for the same reasons. The size of our training sample is small, so the noise in gene expression is still a dominant factor, making the NN easily go overfitting. We choose to reduce the hyper-parameters and training epochs to limit this problem, but it can still be presented by the different *p*-values in Figures 6C, D. Meanwhile, the difference between training sample and test sample ---- the distribution bias ---- is also a known problem for NN, as it has limited power in extrapolations. This could be even worse when NN do not have any a priori knowledge, such as Cox regression model being assumed in the Cox analysis. So, we do not treat this NN results as our fiducial prognosis analysis, even it has stronger significance in risk estimation. We leave more detailed tuning, such as network structure, changing learning rate, optimizer, priors (L1/L2 regularization), etc., for future studies with larger dataset.

Nonetheless, we find PCA can also enhance and subtract the key biological information that is correlated with COAD cancer stages, shown in Figure 7. We find no significant correlation between any individual SIGLEC with a list of clinical features, including age, gender, cancer stage, N-stage, M-stage, and T-stage. However, when applying PCA, we find PC13 is strongly correlated with the cancer stage and T-stage, while PC8 is strongly correlated with N-stage. This works as additional proof of the importance of PCA as a tracer for clinical diagnosis.

In Figure 8, we apply a broad search and find 1,254 DEGs that are highly correlated with COAD and 949 genes that are highly correlated with SIGLECs. The overlapped 18 genes can help us further understand the roles of SIGLECs. With PPI (Figure 8B) and associate Venn diagram (Figure 8C), we identify a list of 12 hub genes, i.e., CD22, SIGLEC15, SIGLEC1, SIGLEC6, SIGLEC10, CD33, CXCR5, SIGLEC9, SIGLEC5, FCRL1, TCL1A, and MAG, while the top three genes from any of the ten algorithms are always CD22, SIGLEC-1,15. We tested when a higher threshold of 0.7 is applied for the PPI, most DEGs’ correlation will vanish, leaving only two chains: one with SIGLEC-9,7,1,15, CD22, CXCR5, and another with SIGLEC-5,14. Such a high threshold will disentangle the SIGLEC-DEG connection, therefore losing the COAD context. The associated GO analysis and GSEA analysis are also presented to illustrate the functions of SIGLECs. Based on all the above analyses, we conclude CD22 and SIGLEC-1,15 are the most promising hub genes in COAD based on their significance and special functions.

Moreover, we find very significant (mostly *p* < 0.001) fingerprints of SIGLECs on immune cell abundance, immune checkpoints, and TMB, as shown in Figure 9. With PCA, we find consistent results as in Figure 1 that PC-1,5 are strongly correlated with most immune cells. PCA can help concentrate the complicated correlations into limited PCs for Macrophages M0, M1, M2, Mast cells resting, T cells CD4 memory resting, etc. It can also largely boost the significance of the correlation to some other immune cells, such as B cells memory, Dendritic cells resting, NK cells activated, T cells CD4 naive, T cells follicular helper, etc. This emphasizes the importance of understanding the co-evolution of different features in multi-dimensions. Such mechanism studies will benefit future studies of cancer evolution as well as treatments.

For typical PCA analysis, PCs with least contribution to the total signal is conventionally removed for the purpose of de-noising. However, in this work under the topic of COAD, due to limited sample-size (<500), measurement noises/biases could be more dominant than part of the weak signals, so we do not perform the removal process. For example, we report significant correlations in PC-1,2,5,8,10,11,13,14 in the previous results, while PC12 has the least significant correlations in Figure 9B, which could contain a large fraction from noise that overwhelms weak biological signal (PC14 for example,). PC3, which ranks 3^rd^ in variance, is also very weakly correlated with immune cells comparing with the other PCs. Unlike PC14, PC3 represents an uncorrelated part between SIGLECs and COAD (PC3-MAG/SIGLEC15 correlation as shown in Figure 1D), which we also successfully subtract. With larger dataset in the future, PCA can exhibit a variety of better results.

As an extension to Figure 2B, we explore the multi-dimensional expression of the SIGLECs family by adopting an unsupervised learning algorithm, the SOM, to further investigate the COAD evolutions. Here we note PCA and SOM are both dimensionality-reduction tools, but focusing on different aspects. PCA is an application of linear algebra, providing translations and rotations in 14D in this analysis. However, it prefers data with Gaussian distribution, which is untrue for many complicated biological data, and it cannot keep the distance between two samples unchanged after the transformation. SOM, on the other hand, keeps the distance/similarities unchanged through a non-linear transformation. Thus, it is a better tool for visualization and pattern-search. Consequentially, due to the complication of the SOM algorithm, it normally requires a very large dataset. Hence, we present the SOM results only as a proof-of-concept study.

We show in Figure 10 that SOM can automatically distinguish the normal sample and the tumor sample in higher dimensions, and successfully categorize them into different groups, while showing the similarities/differences between any two different cases. With SOM, we can also capture how the immune cell abundances change associated with the SIGLECs. We find SOM type-7, type-3, type-0 and type-5,6 correspond to differently expressed SIGLECs (Figure 10), together with other feature changes (Figure 11). This confirms both the sensitivity of SIGLECs to COAD and the promising power of SOM to identify substructures due to potential COAD subtypes. We identify four types of immune cells that play the most important roles together with SIGLECs, namely, Plasma cells, Macrophages M0, Macrophages M2, and Mast cells resting. We conclude that SOM is of great importance in precisely identifying the roles of SIGLECs in the future with larger datasets.

We also note due to the fact SOM and the associated K-means results relay on the random initial weights before training the network, the generated U-matrix (Figure 10A) and type indexes (Figure 10B) are not identical in different runs. Still, we emphasize it is the properties of the separated subgroups (Figures 10E, 11) that matters, while the SOM visualizations in Figures 10A, D are not fixed results, but they work as examples (in different runs with different initial random seeds) to tell the differences between normal sample and COAD subsamples.

Generally speaking, SIGLECs are of great importance in various situations, including their systematical changes with COAD, affecting COAD prognosis, co-evolve with other genes in COAD, its strong correlation with immunotherapy, etc. Through the PCA approach, we identified SIGLEC-1,14,15 plays the most important roles in COAD (Figures 1–4), while through the DEG-PPI approach, we identify SIGLEC-1,15 and CD22 are the hub genes (Figure 8). They are generally consistent, validating the important roles of SIGLEC-1,15. We note the main reason for SIGLEC14 being less significant in the PPI is due to its weaker connections to the DEGs. But the key role of SIGLEC14 can be find from other analysis, i.e., strong correlation to COAD (Figure 1), independent information (Figure 2), significance in prognosis (Figure 3), strong correlation to the immune cells (Figure 9) that identified from SOM (Figure 11). Thus, we still consider it as a promising candidate. Overall, we conclude SIGLEC-1,15 and CD22 are the most sensitive ones in the above analysis based on their outstanding behaviors in Figures 1–4, 6–11. The high sensitivity suggests they can be promising biomarkers for COAD treatments. Future investigation of finding the interaction between them and their sialic-glycan ligands is therefore an important direction of research (Sullivan-Chang et al., 2013; Gianchecchi et al., 2021; Laubli et al., 2022; Saini et al., 2023).

We note that, error analysis is of great importance in statistical studies. In this study with public database, we do not have indicators for different sources of errors. But we can separate them into two categories: statistical error (random motions that shift the expressions independently) and systematical error (which bias the data towards a specific direction). We note statistical error will add random noise into the data (for example, artificial measurement error, day/night difference, before/after food), so they will shift the data points in Figure 2B along the SIGLEC1 direction or CD22 direction independently, therefore weaken the correlation coefficient but without changing the center of the distribution. If the current results are already affected by the statistical error, we can only have stronger intrinsic correlations than the current measurements. This kind of statistical errors can be largely suppressed in the future with larger dataset.

On the other hand, we do not deny the probability of systematical errors. For example, the change of SIGLECs in COAD patients might not be directly due to the cancer cells and the associated immune response, but also affected by the complications (such as perforation, sepsis, or fever condition). Current data do not contain enough information to distinguish the systematical errors. But we note systematical errors are normally meaningful processes that physically exist, and they can be separated in the future with larger and more comprehensive dataset. The existence of systematical errors sometimes leads to inconsistent results when different methods/algorithms are applied. But we also did not observe strong inconsistencies in this study.

Besides, we choose SIGLEC15 as a special case to discuss, due to (1) its different behavior in Figure 1; (2) its weak changes in correlation coefficient, as in Figure 2A; (3) its weak, yet significant, correlation to all the immune checkpoints, as in Figure 9C; (4) its unique behavior in SOM categorizations with type-4,5,6 in Figure 10E. The investigation of SIGLEC15 as a potential target in cancer immunotherapy, highlighted by Wang’s insights (Wang et al., 2019; Sun et al., 2021), opens a promising pathway for advancing diagnostic and therapeutic approaches in COAD (Sun et al., 2021). We argue the role of SIGLEC15 could be important in COAD and require further exploration in the future.

The integrated role of SIGLEC in immune cells, immune checkpoints, and cancer pathways may be a key aspect of its involvement in tumor immune regulation. SIGLEC’s ability to activate Hormone ERE and MT cancer pathways suggests its involvement in signaling pathways associated with cancer. This activation may lead to promoting tumor cell proliferation, migration, and survival, thus facilitating cancer development. The positive correlation of SIGLEC with immune checkpoints indicates that SIGLEC may co-regulate the tumor microenvironment with key factors in immune regulation. This could involve SIGLEC modulating immune evasion strategies. This integration could be realized through SIGLEC-mediated cell signaling networks involving multiple interconnected molecules and pathways. In summary, SIGLEC, through its coordinated regulation of immune cells, immune checkpoints, and cancer pathways, participates in immune evasion and tumor development. These associations provide new research directions for future immunotherapy and cancer biology studies, offering clues to uncover the exact mechanisms of SIGLEC in tumors. Further experimental validation and in-depth molecular biology research are needed to unveil these complex immune regulatory networks.

In the future with larger datasets and better data quality, we expect other machine learning algorithms can play a more important role in a similar analysis. For example, a larger dataset can enable us to further use neural networks to connect SIGLEC with all kinds of features explored in this work. By taking the partial derivative with the neural network, we can directly get the sensitivity of those features to the SIGLECs. This can open a window for finding the optimal biomarker for different types of patients. Additionally, image processing tools such as convolutional neural networks (CNN) can be used directly on the cells (similar to Figure 11) to subtract more complicated information beyond only the expressions of SIGLECs. The co-evolution between amount (SIGLECs expressions, immune cell abundances, etc.) and morphology (cell/structure shape) can be directly connected with such advanced techniques.

## 5 Conclusion

In this work, we explore multiple effects on how SIGLECs co-evolve with COAD, in terms of expressions, prognostic behaviors, clinical trends, enrichment analysis, immune mechanisms, etc. We find SIGLEC family, especially SIGLEC-1,14,15 and CD22, are promising tracers for COAD, while SIGLEC-1,15 and CD22 are identified as hub genes. Beyond conventional single gene-feature analysis, we present high-dimensional analysis with PCA, SOM, NN, LASSO and K-means. We demonstrate PCA is a very powerful tool, which can subtract and enhance essential biological information, especially in COAD prognosis and cancer stages. We visualize the different COAD patterns with SOM and reveal the potential evolutional signals of COAD, which could correspond to COAD subtypes. We emphasize its great potential in finding the potential evolutional paths of COAD in the future with larger datasets.

## Data Availability

The original contributions presented in the study are included in the article/Supplementary Material, further inquiries can be directed to the corresponding author.
