# Supplementary material for: Unveiling the hub genes in the SIGLECs family in colon adenocarcinoma with machine learning
Source: Front Genet. 2024 Apr 8;15:1375100. doi: 10.3389/fgene.2024.1375100 (PMC11033367; doi:10.3389/fgene.2024.1375100)

Supplementary Material


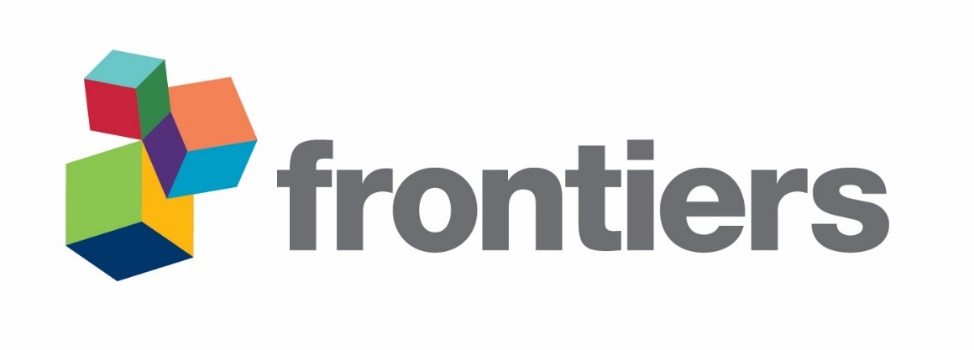


**Supplementary Figure 1.** Flowchart of our study.


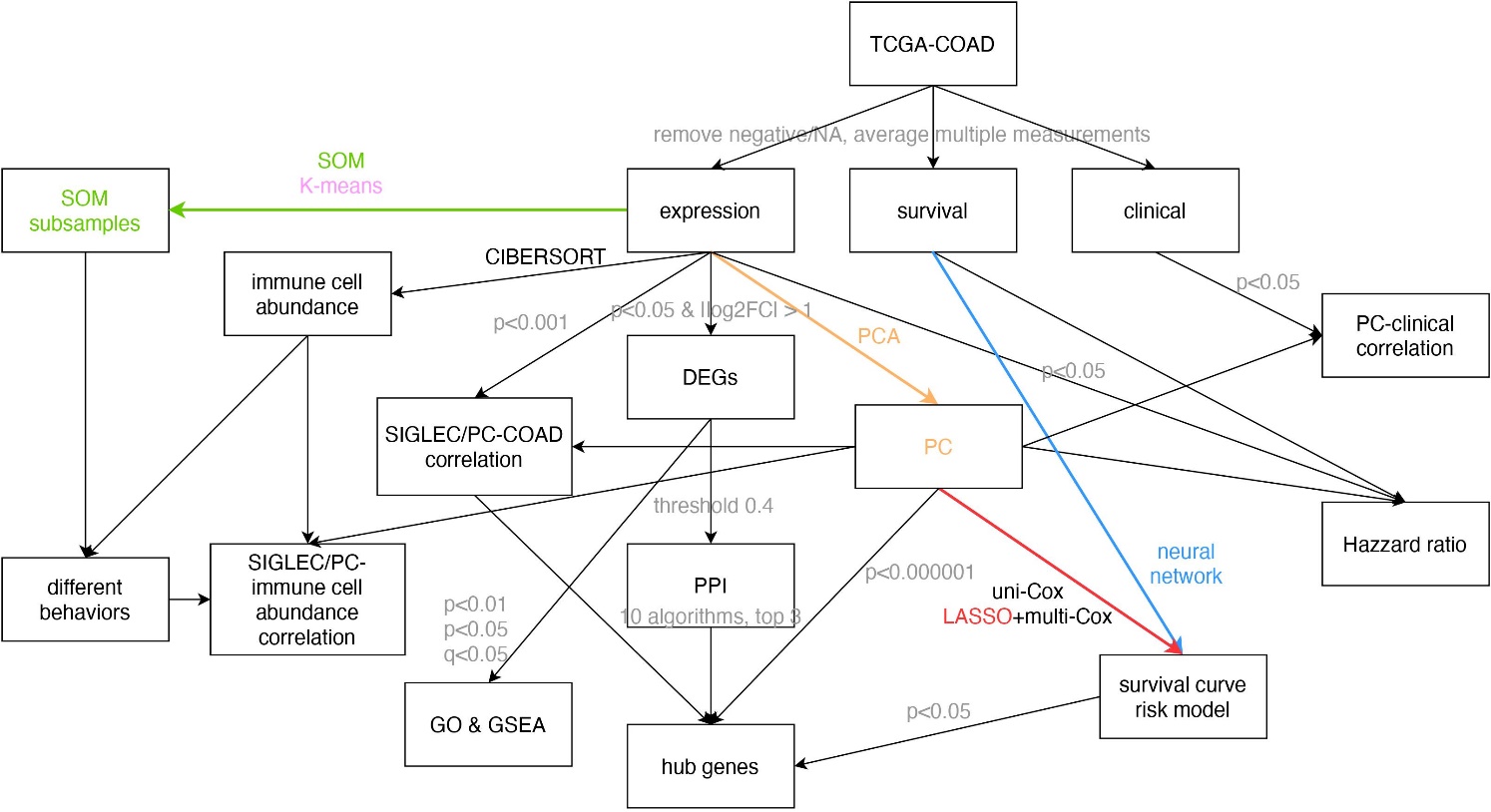


**Supplementary Figure 2.** Expression of SIGLECs Across Various Tumor Types. The illustration depicts the diverse expression patterns of SIGLECs in 20 distinct cancer types compared to their expression levels in normal tissues, as determined from TCGA data. The col-or-coded gradient ranges from green to red, symbolizing the fold change in expression. Green denote down-regulation, while red indicate up-regulation.


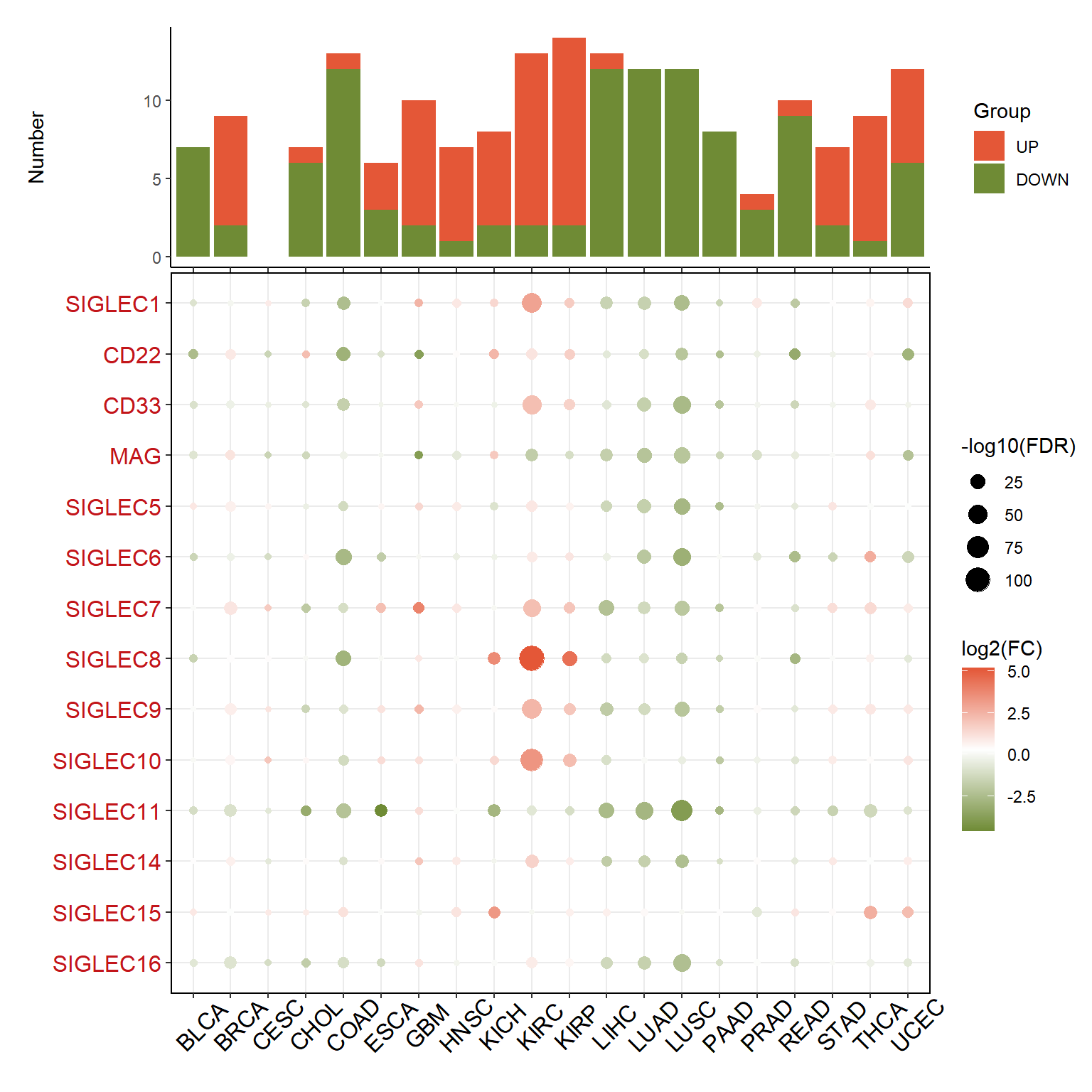


**Supplementary Figure 3.** Survival Curves for the SIGLECs (except for SIGLEC1 shown in Figure 3A) in association with patients’ OS in COAD. Red represents the high SIGLEC-expression group, and cyan represents the low-expression group.


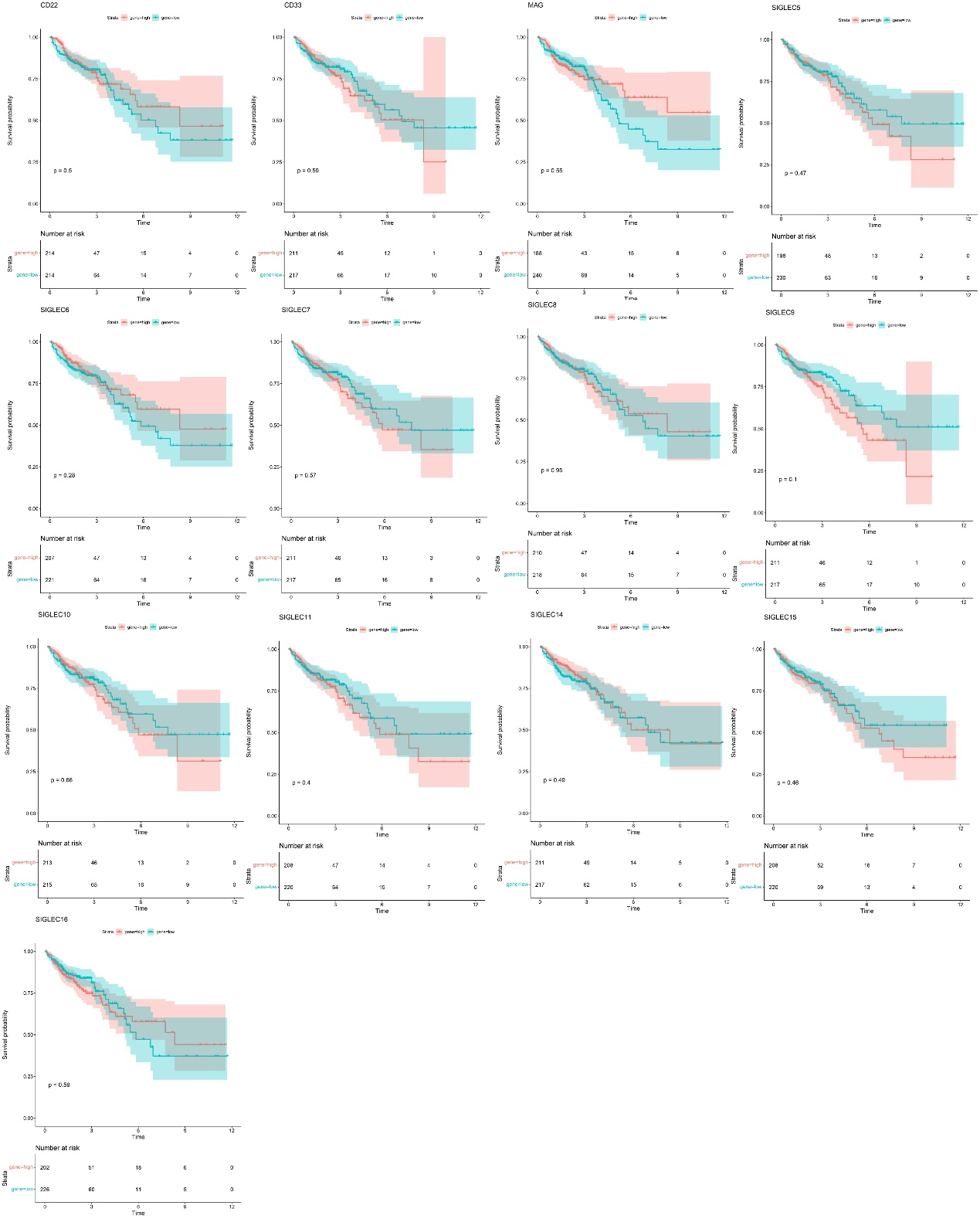


**Supplementary Figure 4.** Survival Curves for the SIGLECs (except for SIGLEC8 shown in Figure 3B) in association with patient’ DFI in COAD. Red represents the high SIGLEC-expression group, and cyan represents the low-expression group.


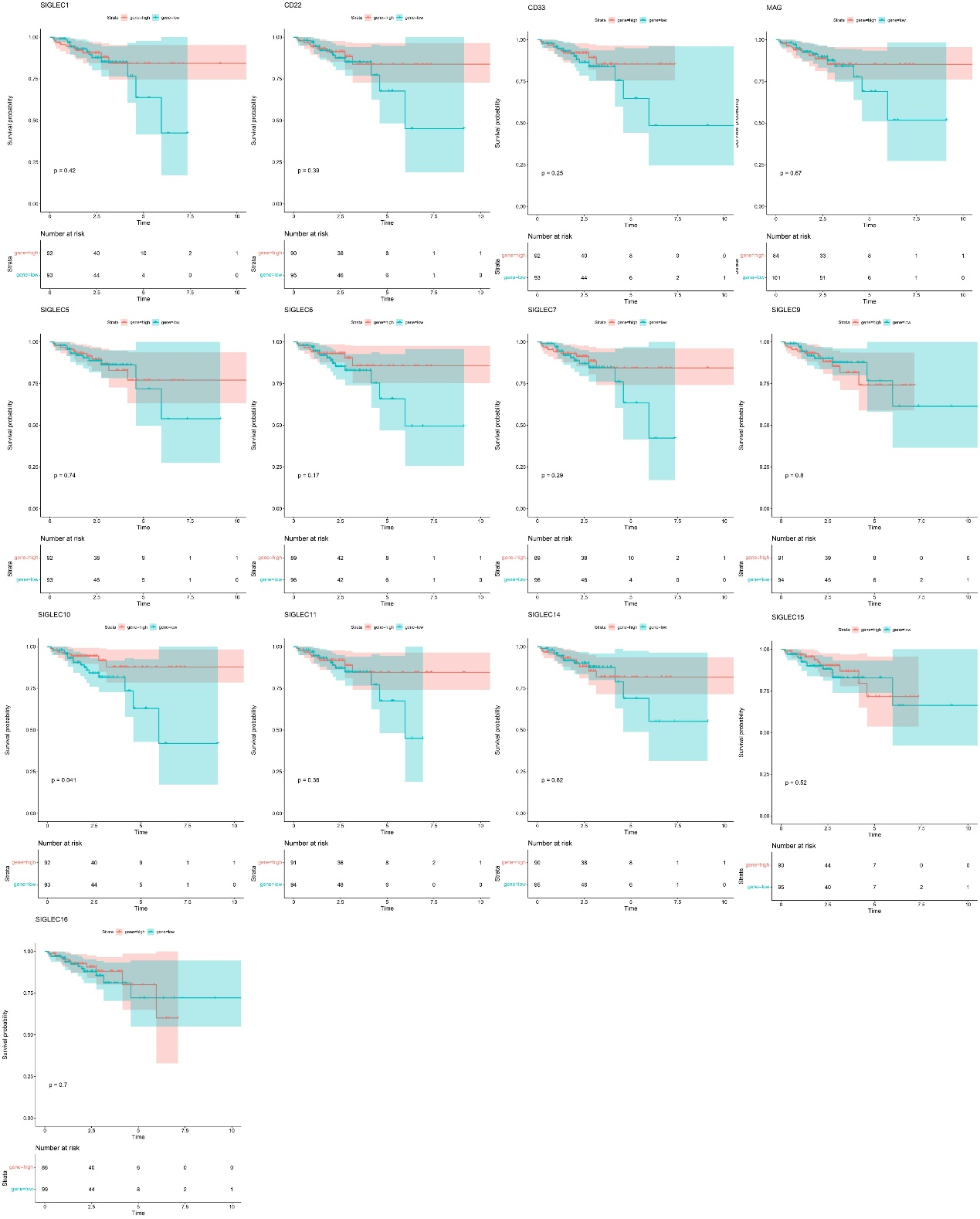


**Supplementary Figure 5.** Survival Curves binning for the SIGLECs.


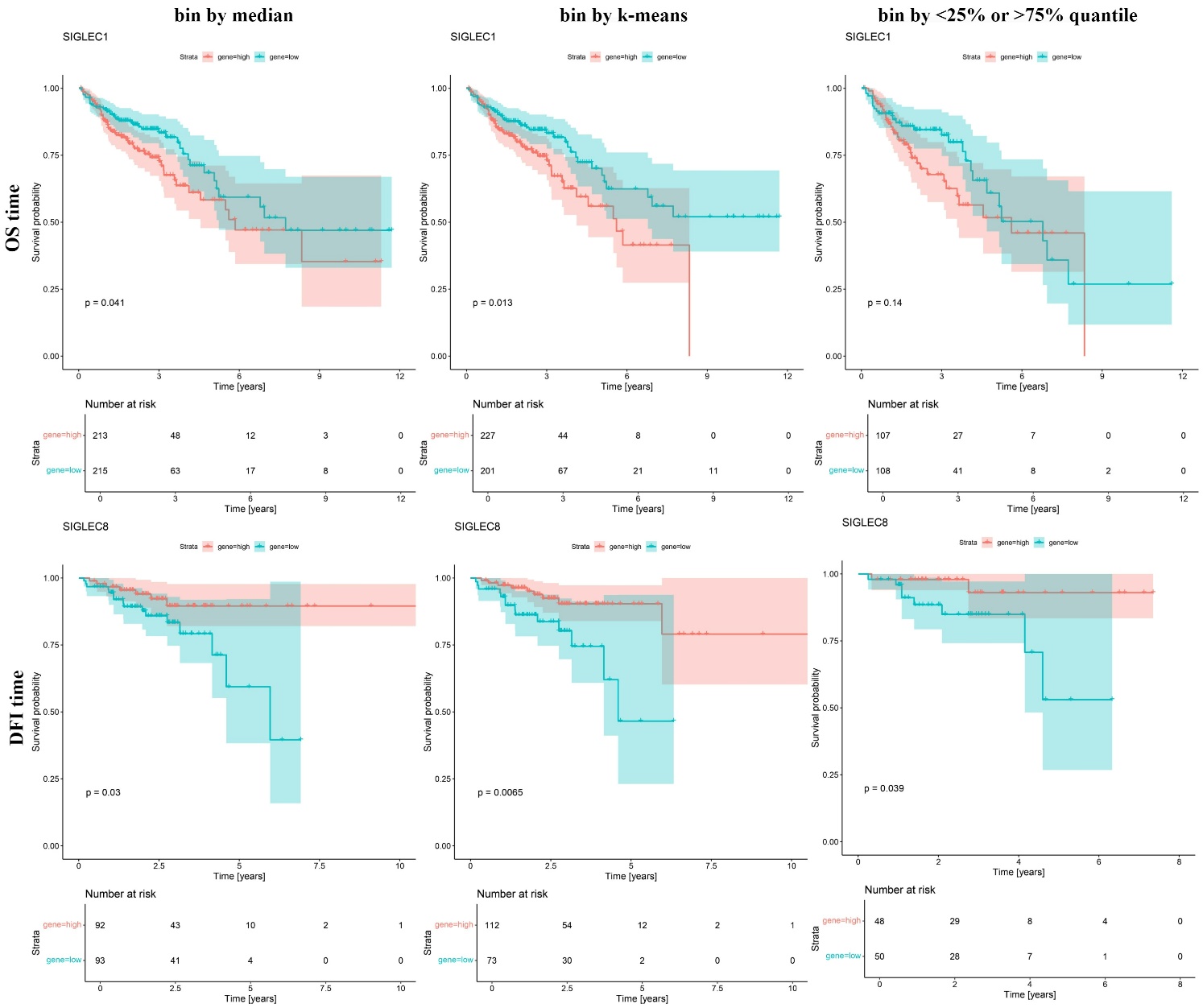


**Supplementary Figure 6.** Evaluating the significances (** for p<0.01, * for p<0.05) of SIGLECs (or PCs) in COAD with different clinical features. (A) SIGLEC v.s. Stage correlation; (B) PC v.s. Stage correlation; (C) SIGLEC v.s. N-stage correlation; (D) PC v.s. N-stage correlation; (E) SIGLEC v.s. T-stage correlation; (F) PC v.s. T-stage correlation.


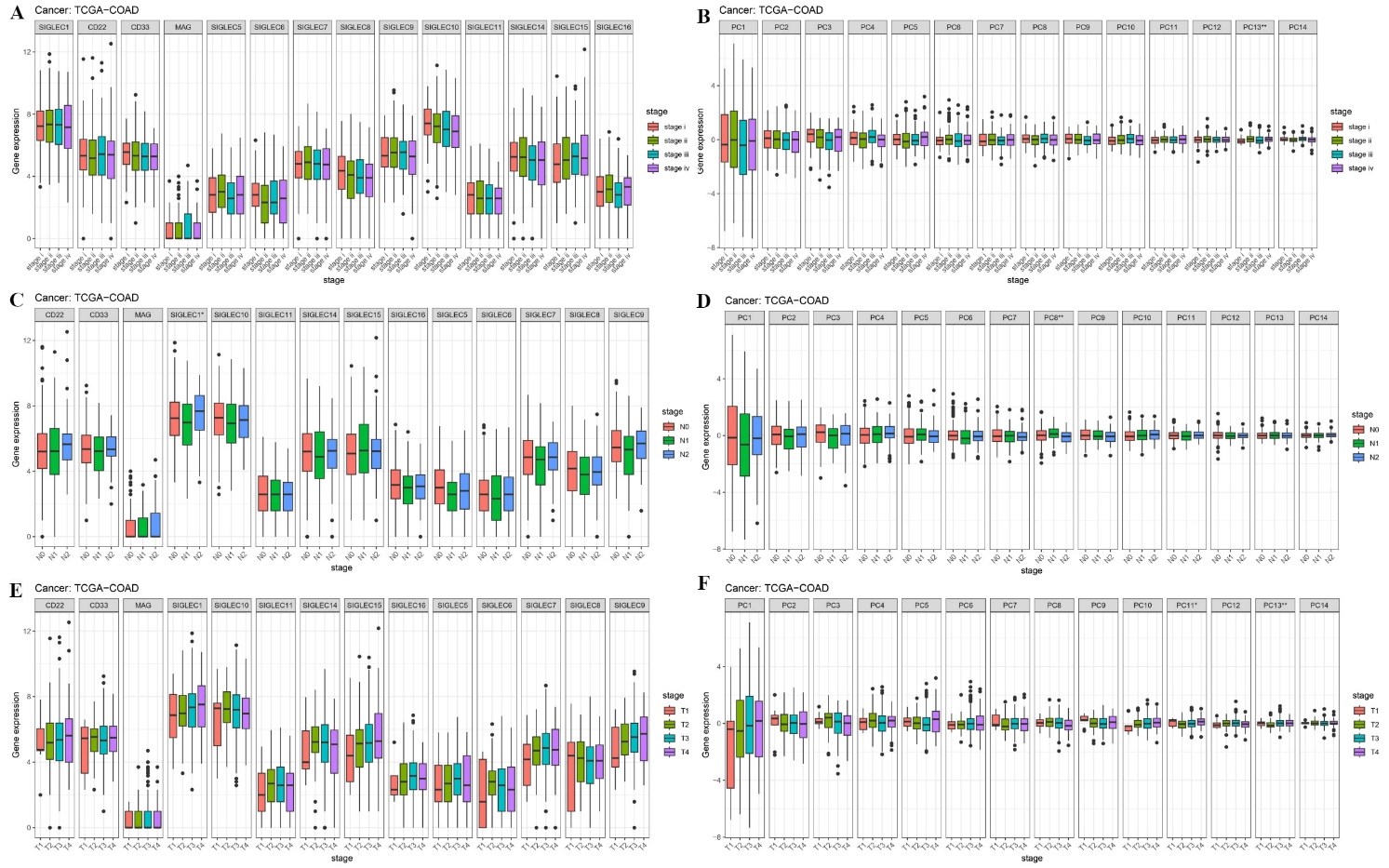


**Supplementary Figure 7.** Single-gene GSEA enrichment results for the SIGLECs (except for SIGLEC1 shown in Figure 8E).


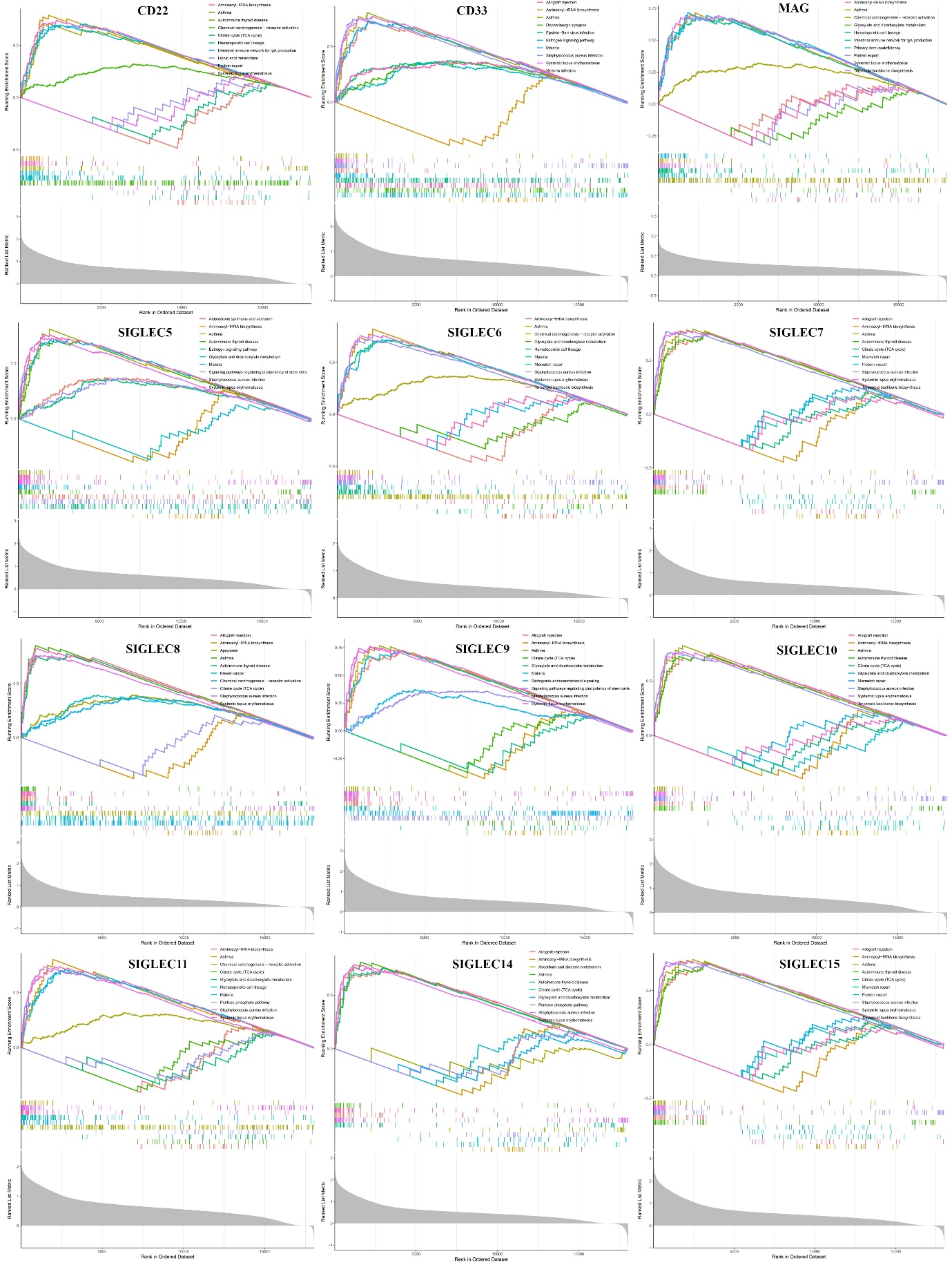


**Supplementary Figure 8.** SIGLECs difference analysis boxplot, with GSE110224/GSE39582 dataset.


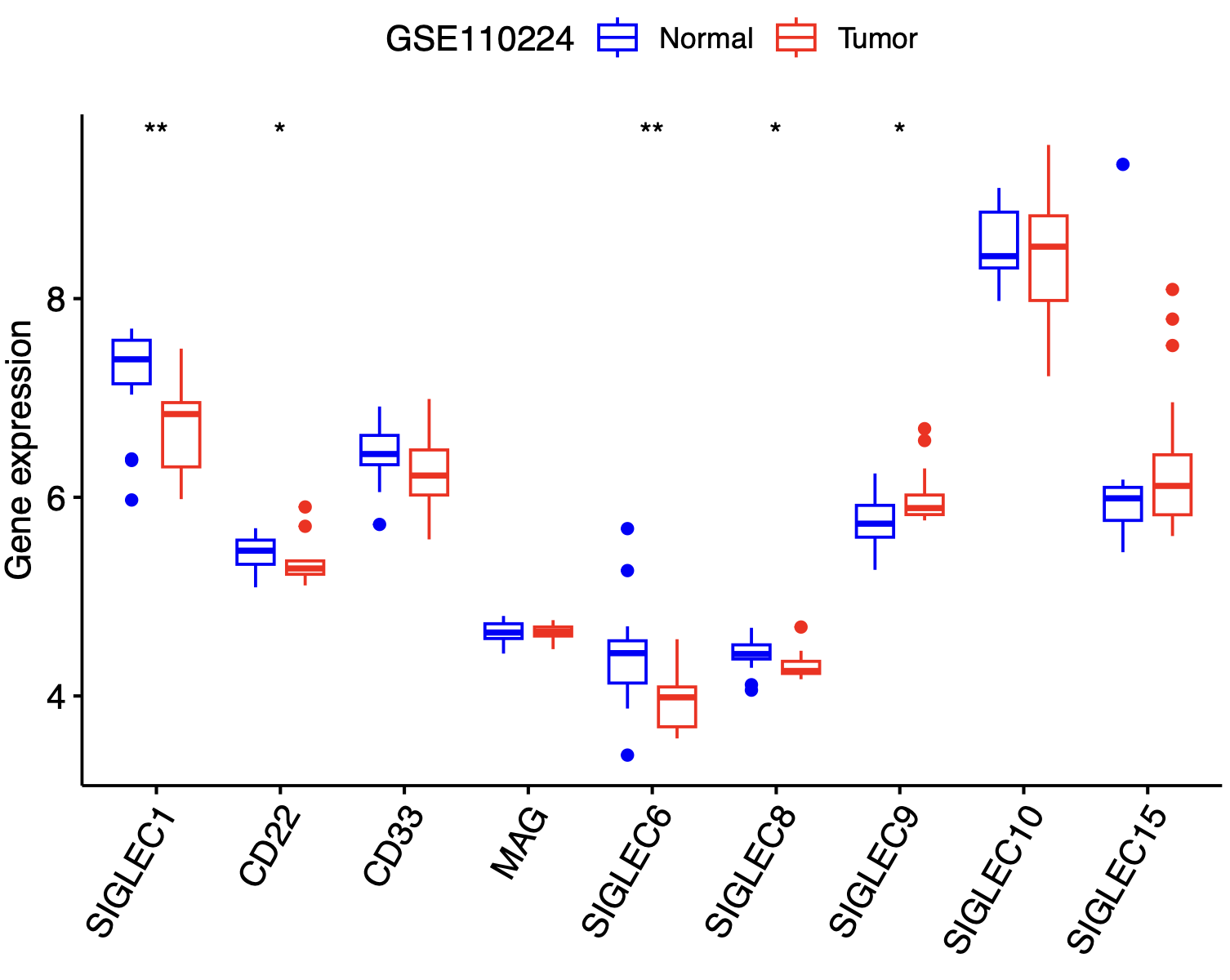


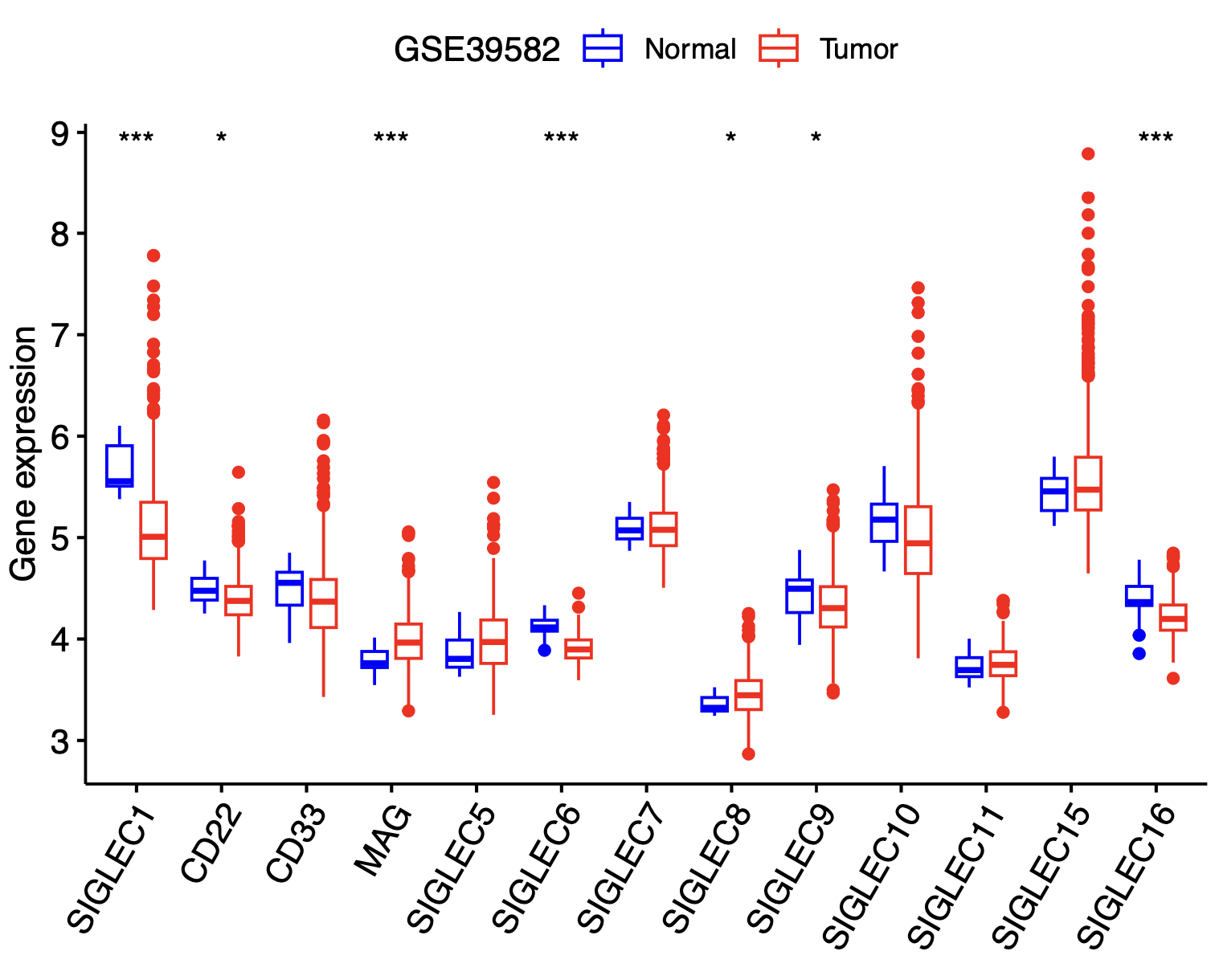

Supplement: Supplementary file 1 [file Table1.DOCX]
